# Influence of dehydroepiandrosterone sulphate levels on the slower age-related decline in grey matter in younger women with polycystic ovary syndrome

**DOI:** 10.1093/braincomms/fcaf052

**Published:** 2025-02-05

**Authors:** Mei-Jou Chen, Chang-Le Chen, Yu-Yuan Chang, Chu-Chun Huang, Wen-Chau Wu, Hong-Nerng Ho, Wen-Yih Isaac Tseng

**Affiliations:** Department of Obstetrics & Gynecology, National Taiwan University Hospital, Taipei 100, Taiwan; Livia Shang Yu Wan Chair Professor of Obstetrics and Gynecology, College of Medicine, National Taiwan University, Taipei 100, Taiwan; Department of Bioengineering, University of Pittsburgh, Pittsburgh, PA 15213, USA; Department of Obstetrics & Gynecology, National Taiwan University Hospital, Taipei 100, Taiwan; Department of Obstetrics & Gynecology, National Taiwan University Hospital, Taipei 100, Taiwan; Institute of Medical Device and Imaging, College of Medicine, National Taiwan University, Taipei 100, Taiwan; Department of Obstetrics & Gynecology, National Taiwan University Hospital, Taipei 100, Taiwan; Research Center for Cell Therapy and Regeneration Medicine, Taipei Medical University, Taipei 100, Taiwan; Institute of Medical Device and Imaging, College of Medicine, National Taiwan University, Taipei 100, Taiwan; AcroViz Inc., Taipei 104, Taiwan

**Keywords:** brain ageing, dehydroepiandrosterone sulphate, obesity, polycystic ovary syndrome, microstructure of brain

## Abstract

Polycystic ovary syndrome (PCOS) is characterized by excess androgens, ovulatory disorders and a higher prevalence of obesity and metabolic disturbances including Type 2 diabetes, hyperlipidaemia and hypertension, some of which are risk factors for neurodegenerative disorders such as Alzheimer’s disease and brain atrophy. However, it is unclear whether brain ageing occurs more rapidly in women with PCOS compared with those without PCOS. Except for the hypothalamic–pituitary–gonadal axis involved in the conventional ovulatory process, little is known regarding the role of the grey matter in the pathogenesis of PCOS, and limited existing studies examining brain structures in PCOS have shown inconsistent results. This case–control study aimed to investigate the age-related differences in total and regional brain grey matter volume and average cortical thickness in young women with and without PCOS by using brain magnetic resonance imaging to understand whether women with PCOS exhibit distinctive patterns of brain ageing, and their association with factors including obesity, hyperandrogenism and metabolic disturbances. Seventy-six women diagnosed with PCOS and 68 age-matched women without PCOS (aged 20–35 years) underwent brain magnetic resonance imaging to measure grey matter volume and cortical thickness. Anthropometric, hormonal and metabolic measurements were conducted to assess their associations with the investigated brain structures. In women without PCOS, increasing age was significantly correlated with a decrease in global grey matter volume (*r* = −0.5598, *P* < 0.0001), while this association was not significant in women with PCOS (*r* = −0.1475, *P* = 0.204). The decline in grey matter volume with age differed significantly between the two groups regardless of obesity (body mass index exceeding 25 kg/m^2^), especially in the frontal, parietal, occipital and temporal regions. After adjusting for dehydroepiandrosterone sulphate (DHEAS) levels, the negative association between age and global grey matter volume became statistically significant in women with PCOS. Increasing age was also significantly associated with a decrease in global cortical thickness in women without PCOS, but not in women with PCOS. Such negative association between global cortical thickness and age was particularly stronger in women with obesity compared with those without. The negative association between age and global cortical thickness in women with PCOS became pronounced after adjusting for DHEAS levels. Women with PCOS experience a milder grey matter loss with age compared with women without PCOS. The neuroprotective effect of high DHEAS levels in women with PCOS may be implicated in this relationship.

## Teaser

Chen *et al*. report that women with polycystic ovary syndrome display a unique brain ageing pattern, featuring a milder decline in grey matter volume and cortical thickness, with dehydroepiandrosterone sulphate levels influencing this pattern. This offers novel insights into polycystic ovary syndrome-related neurobiology and ageing processes.

## Graphical Abstract

**graphical abstract:**
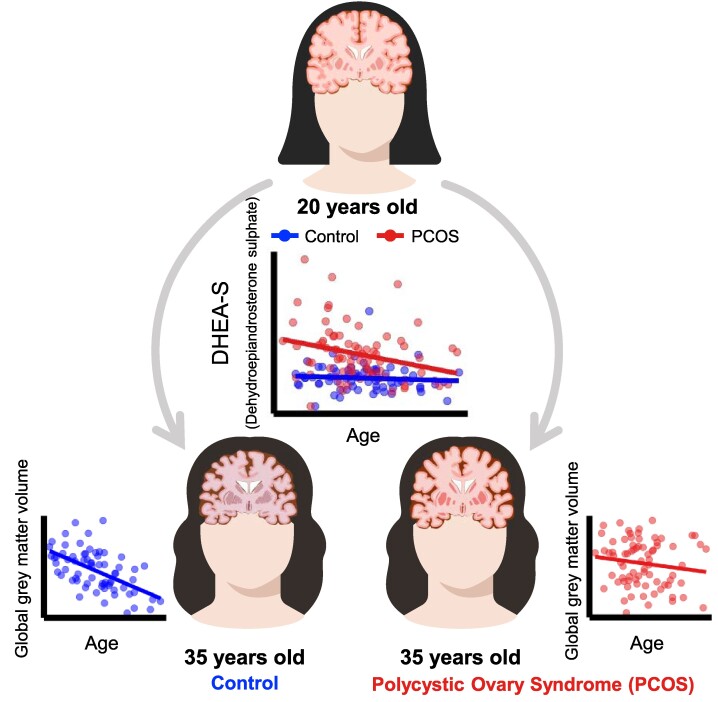

## Introduction

Polycystic ovary syndrome (PCOS) is one of the most common endocrine disorders in women of reproductive age. It is characterized by excess androgen levels, a higher prevalence of metabolic disturbances (e.g. obesity, insulin resistance and high blood pressure) and emotional disorders.^1,2^ Except for the hypothalamic–pituitary–gonadal axis, which is involved in the conventional ovulatory process, little is known regarding the role of other brain regions such as the grey matter in the pathogenesis of PCOS.^3,4^ Although some studies have demonstrated the difference of brain function and connectivity between women with and without PCOS using relatively small samples,^5-7^ few studies have investigated how brain structure relates to the diverse phenotypes in women with PCOS.

Neuroimaging studies using brain MRI have become a tool to identify not only the brain pathology but also as a way to measure metrics that can be compared to other growth charts for anthropometric traits such as weight and height. In a recent study that analysed brain MRI scans of up to 101 457 participants to observe the brain structural change across the lifespan, the results showed that the grey matter volume peaks in early adolescence, while the mean cortical thickness reaches its maximum in early childhood; after these peak periods, both total grey matter volume and mean cortical thickness decline with age.^8^ Several health conditions, heavy metal exposures and psychiatric diseases have been reported to exaggerate the deterioration of brain atrophy with age.^9,10^ High body mass index (BMI) and abdominal obesity have been reported as risk factors for brain grey matter atrophy and are negatively associated with the brain volume in general and vulnerable populations.^10-12^ Diabetes and glucose intolerance are also known to accelerate the deterioration of brain integrity by reducing cortical thickness and brain volume in older adults with or without pre-existing psychiatric and neurological conditions.^13,14^ Theoretically, women with PCOS may have a higher risk of brain atrophy and structural changes related to brain ageing due to several associated risk factors such as insulin resistance and the high prevalence of metabolic disturbances known to contribute to brain degeneration. However, no study has yet proven that women with PCOS have a significantly increased risk of neurodegenerative disorders, such as Alzheimer disease, later in life.^15,16^

In addition to the high prevalence of obesity, glucose intolerance and an increased risk of diabetes, women with PCOS, compared with those without the condition, are also characterized by significantly higher circulating levels of gonadotropins, insulin-like growth factor 1 (IGF-1), insulin-like factor-binding protein 1 and androgens such as testosterone and dehydroepiandrosterone sulphate (DHEAS).^17,18^ The persistently elevated gonadotropin releasing hormone (GnRH) pulse frequency and increased luteinizing hormone (LH) secretion may be a proximate cause of PCOS and could be linked to changes in brain structure and function. The pathogenesis of PCOS has been considered as a result of interaction of obesity and androgen excess.^19^ IGF-1, testosterone and DHEAS have been reported to play critical roles in cortical thickness and brain volume during early brain development,^20-22^ and their levels are particularly higher in adolescence and young adults while decreasing with age.^23,24^ IGF-1, dehydroepiandrosterone (DHEA) and DHEAS have neuroprotective effects, and their levels have been reported to be positively associated with the brain grey matter volume and cortical thickness, as well as having beneficial effects on cognition.^22,25,26^ However, as we previously observed regarding the differential effects of DHEAS and testosterone on metabolic disturbances in young women with PCOS,^27,28^ DHEAS and testosterone have also been reported to have paradoxical effects on neuronal development and brain cortical thickness during adolescence.^20^

Although many characteristic phenotypes of PCOS have been reported to influence neuronal development, function and structural changes of the brain, no studies have determined whether women with PCOS are more susceptible to age-related reductions in brain grey matter volume and average cortical thickness. In this study, by using brain MRI, we aim to investigate age-related differences in total and regional brain grey matter volume and average cortical thickness in relation to the presence of obesity, hyperandrogenism and other phenotypes in young women with and without PCOS.

## Materials and methods

### Ethics

The study was conducted in accordance with the Declaration of Helsinki and approved by the Ethic Committee of the National Taiwan University Hospital (NTUH-REC no. 201810116RINC). All subjects who were willing to participate in this study signed the ethical committee-approved informed consents prior to enrolment.

### Participants

All participants, including both PCOS and control subjects, were women enrolled between 14 February 2019 and 3 March 2022. Individuals with a clinical diagnosis of PCOS, aged between 20 and 35 years, were enrolled from the reproductive endocrinology outpatient clinic of the Department of Obstetrics and Gynecology, National Taiwan University Hospital, Taipei, Taiwan. The diagnosis of PCOS was based on the Rotterdam criteria in which at least two of the three criteria were met: (i) oligomenorrhoea and/or chronic anovulation proved by amenorrhoea, <6 menstrual cycles per year or frequent bleeding <21 days but confirmed anovulation by checking weekly progesterone levels, (ii) biochemical (total testosterone >0.77 ng/ml) or clinical hyperandrogenism as hirsutism and (iii) polycystic ovaries defined as >20 follicles of 2–9 mm diameter per ovary by transvaginal ultrasound or an ovarian volume >10 ml per ovary by transabdominal ultrasound with a distended bladder. All women with PCOS had persistent above-mentioned symptoms for at least 3 years and had not received any medical treatments that might affect the hypothalamic–pituitary–ovarian axis or metabolism such as oral contraceptives, ovulation induction, anti-androgens, anti-obesity and anti-diabetic medications for at least 6 months before enrolment. The exclusion criteria also included a prior or current history of neurological and/or major psychiatric illness, major medical conditions such as cancer, cerebrovascular and cardiovascular diseases, as well as current use of hypnotics, substance abuse and alcohol abuse. Before diagnosing a woman with PCOS, tests for adrenocorticotropin hormone), cortisol, 17-hydroxyprogesterone, thyroid-stimulating hormone and prolactin were conducted to exclude other endocrine disorders that could also cause chronic anovulation and hyperandrogenaemia such as congenital adrenal hyperplasia, adrenal disorders, hypo- or hyperthyroidism and hyperprolactinaemia. Participants with incidental findings on brain MRI were also excluded from the analysis. Consequently, three out of 79 recruited women with PCOS were not analysed, including one with a non-functioning pituitary tumour, one with a cystic lesion in the temporal fossa and one with grey matter heterotopia.

Healthy female volunteers matched for age and BMI were recruited as controls at a 1:1 ratio. The recruitment of study subjects was followed by the enrolment of the control group. The control subjects were selected based on a BMI within the same 5 kg/m^2^ range and 3-year age intervals as participants in the study group. Control subjects only included women with regular menstrual cycles with intervals between 28 and 32 days, no signs of polycystic ovaries and hyperandrogenism such as hirsutism, alopecia and severe acne, no personal history of metabolic, cardiovascular or neurological diseases, as well as no family history of PCOS. They were recruited through advertisements among students and staff within the College and Hospital of National Taiwan University. Questionnaires such as Beck Anxiety Inventory (BAI) and Beck Depression Inventory (BDI) were used to assess emotional disorders. Evaluations involving past medical history, physical examination, pelvic ultrasound and metabolic and hormonal assessments (including androgens, ovarian, pituitary, thyroid and adrenal function) were performed for both control and study subjects to determine their overall health status. Of the 95 volunteers recruited for the study during screening, 22 subjects were excluded before undergoing brain MRI examination due to abnormal blood test results, mostly asymptomatic hyperandrogenaemia. Sixty-eight controls remained in the final analysis since additional five control subjects were excluded due to incidental findings such as brain tumours, non-functioning pituitary hyperplasia, grey matter heterotopia or poor image quality on MRI.

To investigate the effect of obesity, all enrolled subjects were divided into two groups: BMI < 25 and BMI ≥ 25 kg/m^2^ according to the cut-off level recommended for the adult Asian population by WHO expert consultation.^29^

### Anthropometric measurements and biochemical assays

Blood pressure, height, weight, waist and hip circumferences were measured at the time of enrolment according to our previously published protocol.^30^ Data of anthropometric measurements, overnight fasting blood samples and pelvic ultrasound were conducted and collected during the early follicular phase for study and control subjects with spontaneous ovulatory cycles, and randomly for women with PCOS and amenorrhoea. The process of blood sampling and collection has been described in detail in our previous studies.^27,30^ Briefly, blood was processed within 30 min of collection. Blood glucose and insulin samples were stored at 4°C and analysed on the day of sampling. Serum and plasma were aliquoted and frozen at −70°C until assayed. Fasting glucose, total cholesterol, high-density lipoprotein cholesterol (HDL-C), low-density lipoprotein cholesterol (LDL-C) and triglycerides were assayed using a Beckman Coulter automated chemistry analyser (Au5800, Beckman Coulter Ireland Inc.). Serum follicle-stimulating hormone, LH, oestradiol (E2) and progesterone levels were measured using electrochemiluminescence (Elecsys 2010; Cobas e 601, Roche Diagnostics). Anti-Müllerian hormone (AMH) levels were measured with Access 2 Immunoassay system (Beckman Coulter). Serum sex hormone-binding globulin (SHBG), testosterone, DHEAS and insulin levels were measured by immunoassay with Abbott ARCHITECT PLUS (Abbott Laboratories). The homeostasis model assessment of insulin resistance (HOMA-IR) and free androgen index (FAI) were calculated according to the following equations: HOMA-IR = (glucose (mg/dL) × 0.05551) × insulin (μU/ml)/22.5^31^ and FAI (%) = testosterone (ng/ml) × 3.47 × 100/SHBG (nmol/l).^32^ The intra- and inter-assay coefficients of variation of the aforementioned assays were all <10%.

### MRI image acquisition and processing

All MRI data used in this study were acquired on a 3-T MRI system (TIM Trio, Siemens, Erlangen, Germany) with a 32-channel phase array head coil at the National Taiwan University Hospital. Specifically, T_1_-weighted imaging employed magnetization-prepared rapid gradient echo (MPRAGE) sequence with the following imaging parameters: repetition time/echo time = 2000/3 ms, flip angle = 9°, field of view = 256 × 192 × 208 mm^3^ and voxel size = isotropic 1 mm^3^. Following the acquisition of the data, these images were reviewed by an experienced radiologist who was blind to the subject status.

Prior to data analysis, quality assurance (QA) procedures were implemented for all T_1_-weighted images using the Computational Anatomy Toolbox 12 (CAT12),^33^ a retrospective QA framework that quantitatively evaluates key image quality measures. The CAT12 toolbox automatically assessed noise, inhomogeneity and resolution, converting the results to quality ratings. Images were required to achieve a ‘good’ quality rating to be included. Further visual inspection was also conducted on the images to check for any remaining artefacts, excessive motion or abnormal lesions.

To extract effective grey matter features from structural images, voxel-based morphometry (VBM) and surface-based morphometry (SBM) were applied to the 3D MPRAGE images. The image analyses were performed by using CAT12.^33^ For VBM, we estimated regional grey matter volume following the procedures as follows: (i) the structural images were spatially segmented into three major tissue classes including grey matter, white matter and the cerebrospinal fluid, (ii) the segmented tissue maps were spatially registered onto the template in the standard Montreal Neurologic Institute (MNI) space by using geodesic shooting registration algorithm,^34^ (iii) the spatially normalized grey matter images were further modulated with Jacobian determinant maps to preserve the information of individual volumetric measures of grey matter and (4) the well-established probabilistic neuroanatomical atlas, LPBA40, was employed to sample and estimate regional grey matter volume in 56 regions of interest.^35^ For SBM, we used the automated surface-specific pre-processing algorithms established in CAT12 that provided parallel reconstruction of cortical thickness of bilateral hemispheres by using the projection-based approach.^36^ In practice, cortical thickness measures were calculated by estimating the white matter distance given the segmented tissue maps in the native space. The distance and the derived neighbour relationship were utilized to project local maxima (representing the thickness) onto other voxels of grey matter. This method embedded partial volume correction and adjustments for sulcal blurring and asymmetries. After the initial reconstruction of grey matter surface, topological defects were removed using a spherical harmonics approach.^37^ Following by a surface refinement to create the final central surface mesh, the individual cortical surface mesh was reparameterized and spatially registered to the surface-based standard template by using a spherical mapping method^38^ with minimal spatial distortions. After the surface reconstruction, the Desikan–Killiany^39^ cortical atlas with 68 regions of interest in the standard template space was transformed to sample average cortical thickness on a regional basis in the native space. Through these two morphological analyses, 56 volumetric features and 68 cortical thickness features were obtained to represent grey matter anatomical features.

### Statistical analysis

All measurements were presented as mean with standard deviation (in parentheses) unless indicated otherwise. Two-way ANOVA was applied to evaluate the influence of PCOS or age-matched control status and of being obese or non-obese, as well as to determine the possible interaction between these variables, on brain structures and phenotypic variables. Multiple linear regression with a group-by-age interaction term was used to examine whether the decline in overall cortical thickness and overall grey matter volume with age differed between women with and without PCOS. Multiple comparisons for neuroanatomical measures were addressed by using Bonferroni’s method for mass univariate tests and *post hoc* comparisons in the regression analysis to test specific neuroanatomical lobes and BMI-specific groups. Exploratory correlation analysis and partial correlation analysis were used to determine the correlation between regional brain features and phenotypic variables with and without adjustment for levels of BMI and DHEAS. Stepwise multiple linear regression selected by Akaike information criterion value was performed using total grey matter volume and cortical thickness as dependent variables and all the phenotypic variables including age as independent variables. All of the plots were generated through ggplot2 R package,^40^ and the statistical analyses were performed using R (version 4.2.1) and R Studio (version 2022.02.3+492.pro3) software. For exploratory analysis, an uncorrected alpha of 0.05 was used as significance level; otherwise, the *P-*values considered statistically significant after multiple comparison correction for neuroanatomical measurements were indicated.

## Results

### Demographic information

The clinical characteristics of women with and without PCOS are presented in Table 1 and Supplementary Table 1. There was no significant difference in age between the two groups. As expected, obesity contributed significantly to higher values of BMI, insulin, glucose, HOMA-IR, blood pressure, triglycerides and FAI and lower levels of HDL-C and SHBG. Additionally, beyond metabolic factors related to insulin resistance and dyslipidaemia, PCOS was significantly associated with higher baseline levels of oestradiol (E2), LH, DHEAS, testosterone, AMH, FAI, BAI scores and BDI scores and lower SHBG levels, independent of obesity. The interaction term between PCOS status and BMI levels was statistically significant for diastolic blood pressure, HOMA-IR, total cholesterol, LDL-C and FAI, indicating that the effect of PCOS on these parameters may be influenced by BMI.

**Table 1:** Demographic and clinical characteristics of women with and without PCOS

	PCOS			Control			Two-way ANOVA *P*-value		
	All (*n* = 76)	BMI < 25 kg/m^2^ (*n* = 35)	BMI ≥ 25 kg/m^2^ (*n* = 41)	All (*n* = 68)	BMI < 25 kg/m^2^ (*n* = 45)	BMI ≥ 25 kg/m^2^ (*n* = 23)	Main effect of PCOS	Main effect of BMI	Interaction effect
Age (years)	25.4 (3.4)	24.8 (3.2)	26.0 (3.5)	25.8 (3.4)	25.1 (3.2)	27.3 (3.3)	0.441	0.004*	0.395
BMI (kg/m^2^)	27.0 (6.4)	21.3 (1.8)	31.9 (4.6)	23.6 (4.6)	21.0 (1.9)	28.7 (4.0)	<0.001*	<0.001*	0.012*
SBP (mmHg)	116.7 (13.6)	109.2 (10.2)	123.1 (12.9)	105.8 (9.9)	103.4 (8.7)	110.6 (10.5)	<0.001*	<0.001*	0.07
DBP (mmHg)	77.2 (10.9)	71.6 (7.4)	81.9 (11.2)	71.3 (7.2)	71.2 (7.0)	71.4 (7.6)	<0.001*	<0.001*	<0.001*
Insulin (μU/ml)	10.4 (5.9)	6.0 (2.1)	14.2 (5.5)	8.8 (6.8)	7.2 (3.5)	12.0 (10.0)	0.078	<0.001*	0.074
Glucose (mg/dL)	88.7 (10.2)	84.5 (4.8)	92.2 (12.1)	83.7 (5.9)	82.4 (5.7)	86.2 (5.4)	<0.001*	<0.001*	0.151
HOMA-IR	2.3 (1.4)	1.3 (0.4)	3.2 (1.3)	1.9 (1.5)	1.5 (0.8)	2.6 (2.3)	0.024*	<0.001*	0.041*
Total cholesterol (mg/dL)	197.2 (38.2)	188.9 (29.4)	204.2 (43.5)	180.0 (38.5)	185.3 (42.6)	169.6 (26.8)	0.007*	0.823	0.019*
Triglyceride (mg/dL)	111.3 (66.8)	75.0 (37.4)	142.3 (71.0)	75.0 (54.4)	63.5 (21.1)	97.6 (85.6)	<0.001*	<0.001*	0.084
LDL-C (mg/dL)	122.3 (38.4)	104.9 (29.9)	137.1 (38.9)	104.6 (31.9)	107.2 (35.6)	99.7 (22.8)	0.002*	0.013*	<0.001*
HDL-C (mg/dL)	61.2 (46.6)	66.9 (13.4)	56.2 (62.2)	59.5 (12.8)	62.7 (12.5)	53.2 (11.1)	0.773	0.091	0.919
Testosterone (ng/ml)	0.6 (0.2)	0.6 (0.2)	0.6 (0.2)	0.5 (0.1)	0.5 (0.1)	0.5 (0.1)	<0.001*	0.373	0.303
DHEAS (μg/dL)	10.1 (4.5)	10.5 (5.3)	9.7 (3.9)	6.1 (2.3)	5.8 (1.8)	6.6 (2.9)	<0.001*	0.88	0.216
SHBG (nmol/l)	28.6 (18.0)	41.2 (18.5)	17.9 (7.9)	53.8 (27.0)	64.2 (26.4)	33.4 (13.1)	<0.001*	<0.001*	0.245
FAI (%)	10.0 (6.7)	6.5 (4.4)	13.0 (6.8)	4.1 (2.6)	3.0 (1.1)	6.1 (3.5)	<0.001*	<0.001*	0.026*
AMH (ng/ml)	12.2 (5.8)	13.0 (6.3)	11.4 (5.2)	4.4 (2.5)	4.7 (2.5)	3.7 (2.4)	<0.001*	0.121	0.667
BAI score	7.2 (6.3)	8.0 (7.5)	6.4 (5.1)	4.1 (4.0)	4.5 (4.4)	3.3 (3.0)	<0.001*	0.114	0.854
BDI score	9.1 (8.2)	9.6 (9.0)	8.7 (7.5)	5.7 (6.0)	6.2 (6.1)	4.7 (5.9)	0.006*	0.331	0.81

Data are presented as mean (standard deviation).

AMH, anti-Müllerian hormone; BAI, Beck Anxiety Inventory; BDI, Beck Depression Inventory; BMI, body mass index; DBP, diastolic blood pressure; DHEAS, dehydroepiandrosterone sulphate; FAI, free androgen index; HDL-C, high-density lipoprotein cholesterol; HOMA-IR, homeostasis model assessment of insulin resistance; LDL-C, low-density lipoprotein cholesterol; SBP, systolic blood pressure ; SHBG, sex hormone-binding globulin.

^*^ *P* < 0.05 was considered statistically significant by two-way ANOVA test.

### Grey matter volume

#### Global grey matter volume

**Figure 1:**
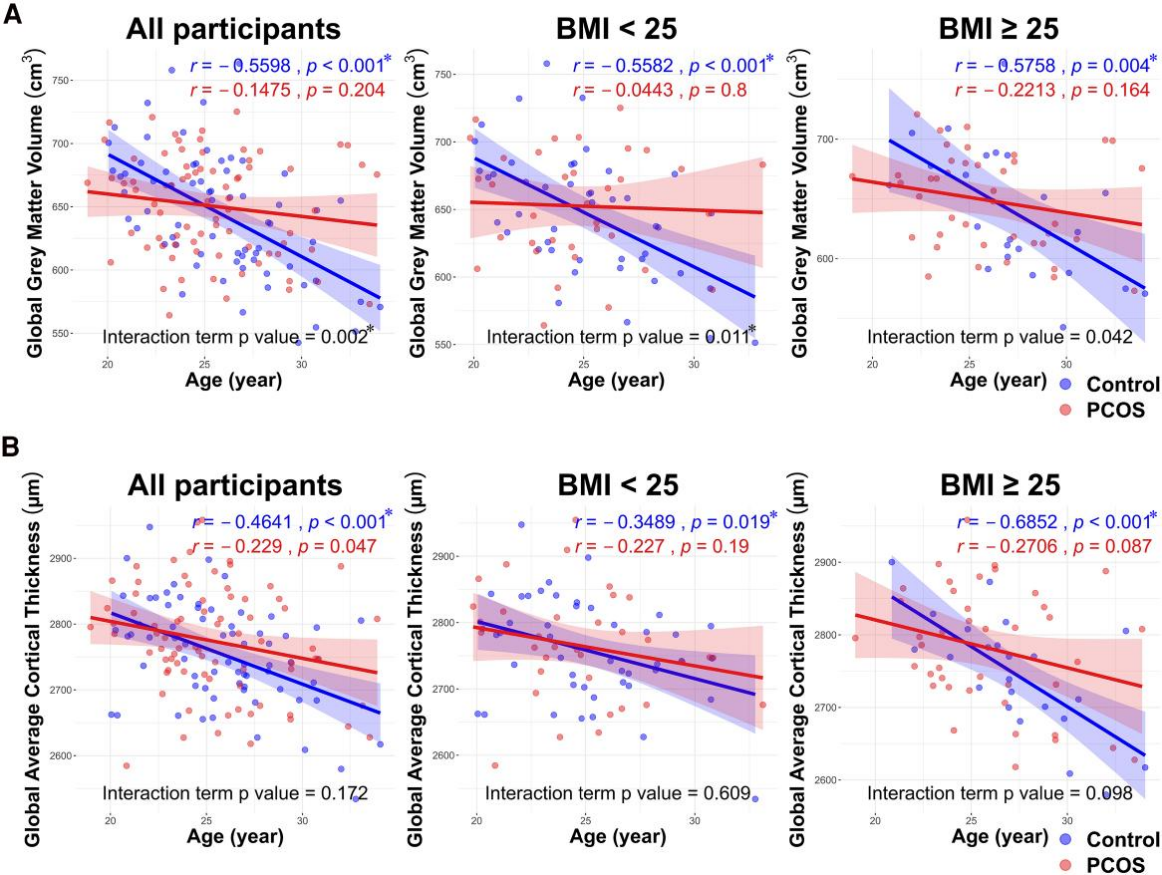
**Distinctive age-related changes in global grey matter volume and global average cortical thickness in women with and without PCOS.** (**A**). It illustrates the age-related changes in total grey matter volume with age in women with and without PCOS. Notably, a significant decline in total grey matter volume is observed with increasing age in women without PCOS, while such decline is not as pronounced in those with PCOS. The statistically significant interaction term defines the distinctive regression lines for the PCOS and non-PCOS groups, highlighting the divergence in brain structural changes associated with ageing in these populations, independent of obesity. (**B**). It demonstrates the age-related changes in average cortical thickness with age in women with and without PCOS. Notably, a significant decline in average cortical thickness with increasing age is observed in women without PCOS, especially among obese women. *r* is the Pearson correlation coefficient; the interaction term is the interaction between age and global grey matter volume or global average cortical thickness in linear regression (control group: *n* = 68, PCOS group: *n* = 76). Bold lines represent the trend lines of age and global grey matter volume or global average cortical thickness, while the shaded areas indicate the 95% confidence intervals. *P < 0.025 was considered statistically significant according to Bonferroni’s correction for multiple comparisons.

The mean global grey matter volume of all enrolled subjects showed no significant difference between women with and without PCOS and between obese and non-obese women (Table 2). However, as shown in Fig. 1A, regardless of obesity, increasing age was significantly associated with a decrease in global grey matter volume in controls, while in women with PCOS, this association was not significant. The decrease in grey matter volume with age differed significantly between the two groups, especially in non-obese women (Fig. 1A). By using univariate analysis to investigate association of global grey matter volume with other clinical characteristics such as anthropometric measurements, hormone levels, metabolic profiles, anxiety scores and depression scores, the results in Table 3 showed that only DHEAS levels revealed significantly negative association with the global grey matter volume in women with PCOS. The negative association between age and global grey matter volume became statistically significant only after adjustment for the DHEAS levels. Furthermore, by using stepwise regression model to select the best fit after considering all the explanatory variables for predicting the decline in global grey matter volume, only age and DHEAS remained in the final model (Table 3). These findings suggest a crucial role of DHEAS level in modulating the age-related decline in global grey matter volume in young women both with and without PCOS. As shown in Supplementary Fig. 1A, as expected, DHEAS levels decrease with age and are higher in women with PCOS compared with those without PCOS. However, the rate of decline in DHEAS level with age is faster in women with PCOS than those without. This may explain why DHEAS levels are positively correlated with global grey matter volume in women without PCOS, but negatively correlated with global grey matter volume in women with PCOS (Supplementary Fig. 1B). As the result, the significant and negative association between age and global grey matter volume in women with PCOS could only be highlighted after adjustment for the DHEAS levels.

**Table 2:** Grey matter volume and cortical thickness of whole and regional brain of women with and without PCOS

		PCOS			Control			Two-way ANOVA *P*-value		
		All (*n* = 76)	BMI < 25 kg/m^2^ (*n* = 35)	BMI ≥ 25 kg/m^2^ (*n* = 41)	All (*n* = 68)	BMI < 25 kg/m^2^ (*n* = 45)	BMI ≥ 25 kg/m^2^ (*n* = 23)	Main effect of PCOS	Main effect of BMI	Interaction effect
Whole brain	Global grey matter volume	650.5 (40.7)	652.6 (41.7)	648.8 (40.2)	644.3 (48.8)	647.1 (46.1)	638.8 (54.3)	0.409	0.453	0.771
	Global cortical thickness	2773.7 (83.2)	2765.0 (81.9)	2781.2 (84.7)	2753.9 (78.8)	2757.8 (78.6)	2746.1 (80.5)	0.146	0.788	0.322
Regional grey matter volume	Frontal	182.2 (13.9)	182.5 (13.6)	182.0 (14.3)	181.4 (14.5)	181.8 (12.9)	180.5 (17.6)	0.716	0.742	0.866
	Parietal	105.1 (7.5)	105.6 (7.9)	104.6 (7.2)	103.1 (10.1)	104.1 (9.6)	101.1 (11.0)	0.168	0.212	0.511
	Occipital	55.1 (4.3)	54.7 (4.5)	55.4 (4.2)	54.7 (5.8)	55.0 (5.5)	54.0 (6.4)	0.629	0.927	0.331
	Temporal	134.3 (8.9)	134.5 (9.2)	134.2 (8.7)	133.1 (10.8)	134.1 (10.5)	131.1 (11.5)	0.452	0.385	0.432
	Insula	11.7 (1.0)	11.7 (0.9)	11.7 (1.0)	11.6 (0.9)	11.6 (0.9)	11.6 (0.9)	0.492	0.95	0.933
	Limbic	24.9 (1.89)	25.0 (1.8)	24.8 (1.9)	24.8 (2.0)	24.9 (1.8)	24.5 (2.3)	0.7	0.42	0.819
	Caudate	7.0 (0.8)	7.1 (0.8)	6.8 (0.7)	6.9 (0.7)	6.9 (0.8)	6.8 (0.7)	0.724	0.088	0.253
	Putamen	7.5 (1.1)	7.4 (1.1)	7.7 (1.2)	7.2 (1.0)	7.1 (1.0)	7.3 (1.0)	0.063	0.143	0.78
	Cerebellum	86.2 (7.4)	87.3 (7.2)	85.2 (7.4)	84.9 (8.2)	84.7 (8.9)	85.3 (6.7)	0.326	0.508	0.302
	Brain stem	1.1 (0.2)	1.2 (0.2)	1.1 (0.2)	1.2 (0.2)	1.2 (0.2)	1.0 (0.2)	0.553	<0.001*	0.43
Regional cortical thickness	Frontal	2660.2 (81.0)	2660.5 (84.7)	2660.0 (78.8)	2635.7 (74.8)	2637.3 (74.1)	2632.6 (77.8)	0.064	0.863	0.876
	Parietal	2422.1 (70.2)	2412.8 (65.7)	2430.1 (73.7)	2393.7 (67.1)	2393.6 (69.3)	2393.9 (64.1)	0.015	0.413	0.475
	Occipital	2076.8 (62.6)	2073.2 (68.0)	2079.9 (58.2)	2065.1 (69.0)	2064.4 (69.4)	2066.4 (69.7)	0.291	0.683	0.835
	Temporal	2770.2 (108.4)	2757.2 (124.0)	2781.4 (93.2)	2746.1 (81.8)	2738.0 (86.0)	2761.9 (71.8)	0.137	0.149	0.993
	Cingulate	2399.8 (75.8)	2400.1 (78.8)	2399.6 (74.1)	2383.6 (78.3)	2378.9 (80.3)	2392.8 (75.2)	0.212	0.657	0.586
	Insula	2967.3 (129.9)	2982.0 (127.0)	2954.7 (132.5)	2966.5 (150.8)	2943.2 (142.5)	3012.1 (159.2)	0.973	0.513	0.046

Data are presented as mean (standard deviation). Bonferroni’s methods were applied for multiple comparison correction of neuroanatomical measurements; therefore, the statistically significance (*) was defined as *P* < 0.025 (0.05/2) for whole brain, *P* < 0.005 (0.05/10) for regional brain volume and *P* < 0.008 (0.05/6) for regional cortical thickness between groups as indicated.

cm^3^, unit of grey matter volume; µm, unit of cortical thickness.

**Table 3:** Univariate linear regression analysis and multiple linear regression with stepwise selection to identified all the variable contribute to global grey matter volume and global cortical thickness

	Global GMV total		Global GMV PCOS		Global GMV control		Global CT total		Global CT PCOS		Global CT control	
	Univariate	Model^a^	Univariate	Model^a^	Univariate	Model^a^	Univariate	Model^a^	Univariate	Model^a^	Univariate	Model^a^
Age	−4.78*** (1.04)	−4.98*** (1.05)	−1.77 (1.38)	−2.945* (1.365)	−8.10*** (1.48)	−7.67*** (1.45)	−8.23*** (1.91)	−9.75*** (1.93)	−5.62* (2.78)	−7.06* (2.74)	−10.85*** (2.55)	−11.78*** (2.50)
BMI	−0.31 (0.64)		0.13 (0.74)		−1.89 (1.28)		1.50 (1.16)	2.83 (1.48)	1.84 (1.50)		−0.50 (2.11)	
SBP	0.02 (0.29)		−0.01 (0.35)		−0.29 (0.61)		0.87 (0.52)		1.05 (0.70)		−0.13 (0.98)	
DBP	−0.05 (0.38)		−0.18 (0.43)		−0.09 (0.84)		0.78 (0.70)		0.95 (0.88)		−0.60 (1.35)	
Insulin	−0.45 (0.59)		−0.12 (0.80)		−0.86 (0.88)		2.26* (1.06)	2.83* (1.27)	2.64 (1.62)		1.64 (1.42)	
Glucose	−0.32 (0.43)		−0.35 (0.46)		−0.80 (1.01)		−0.10 (0.78)		−0.73 (0.95)		0.41 (1.65)	
HOMA-IR	−1.96 (2.50)		−0.80 (3.32)		−3.83 (3.86)		9.09* (4.51)		9.29 (6.71)		7.24 (6.23)	13.61* (5.47)
T-cholesterol	−0.07 (0.10)		−0.09 (0.12)		−0.10 (0.16)		0.16 (0.17)		0.46 (0.25)		−0.28 (0.25)	
Triglyceride	−0.03 (0.06)		0.02 (0.07)		−0.17 (0.11)		0.01 (0.11)		0.14 (0.14)		−0.35 (0.17)	
LDL-C	−0.03 (0.10)		−0.03 (0.12)		−0.08 (0.19)		0.28 (0.19)		0.61* (0.24)	0.57* (0.23)	−0.40 (0.30)	
HDL-C	−0.07 (0.11)		−0.11 (0.10)		0.46 (0.47)		−0.01 (0.20)		−0.10 (0.21)		1.18 (0.75)	
AMH	0.58 (0.75)		−0.48 (1.05)		3.53 (2.37)		−0.06 (1.38)		−0.54 (2.31)		−2.83 (3.88)	
Testosterone	18.11 (23.11)		−13.26 (26.81)		70.60 (45.97)		64.34 (41.94)	102.80* (42.97)	20.10 (54.88)		115.59 (74.26)	
DHEAS	−0.50 (0.90)	−1.45 (0.85)	−2.47* (1.00)	−3.09** (1.02)	5.00 (2.59)	4.52* (2.18)	0.22 (1.65)		−2.93 (2.10)	−4.07 (2.05)	6.53 (4.23)	6.14 (3.68)
SHBG	0.11 (0.14)		0.06 (0.26)		0.29 (0.22)	0.28 (0.18)	−0.16 (0.26)		−0.10 (0.58)		0.10 (0.36)	
FAI	−0.22 (0.63)		−0.41 (0.71)		−2.29 (2.26)		0.57 (1.15)	−3.35* (1.36)	0.02 (1.45)		−3.14 (3.66)	
BAI score	1.27 (0.66)	1.00 (0.62)	0.81 (0.74)		2.32 (1.46)		1.02 (1.22)		−0.33 (1.53)		3.05 (2.37)	
BDI score	0.53 (0.50)		0.29 (0.58)		0.78 (0.99)		0.93 (0.92)		0.258 (1.18)		1.47 (1.60)	
Adjusted *R*^2^		0.1444		0.1077		0.3402		0.1857		0.1412		0.2717

Data are presented as Beta (standard error).

cm^3^, unit of grey matter volume; µm, unit of cortical thickness; AMH, anti-Müllerian hormone; BAI, Beck Anxiety Inventory; BDI, Beck Depression Inventory; BMI, body mass index; CT, cortical thickness; DBP, diastolic blood pressure; DHEAS, dehydroepiandrosterone sulphate; E2, oestradiol; FAI, free androgen index; FSH, follicle-stimulating hormone; GMV, grey matter volume; HDL-C, high-density lipoprotein cholesterol; HOMA-IR, homeostasis model assessment of insulin resistance; LDL-C, low-density lipoprotein cholesterol; LH, luteinizing hormone; SBP, systolic blood pressure; SHBG, sex hormone-binding globulin.

^a^The stepwise selection model considered all the listed variables contributing to global grey matter volume and global cortical thickness, including FSH, LH and E2. However, due to space limitation, the univariate analysis results for FSH, LH and E2 are given in Supplementary Table 2.

***P < 0.001, **P < 0.01, *P < 0.05.

#### Regional grey matter volume

The grey matter volume in specific brain regions and their corresponding gyri is given in Table 2 and Supplementary Table 3. The grey matter volume of the left postcentral gyrus in the parietal region was significantly higher in women with PCOS than those without PCOS. Obesity, rather than PCOS, was significantly associated with lower grey matter volume in the brain stem regions across all enrolled women.

**Figure 2:**
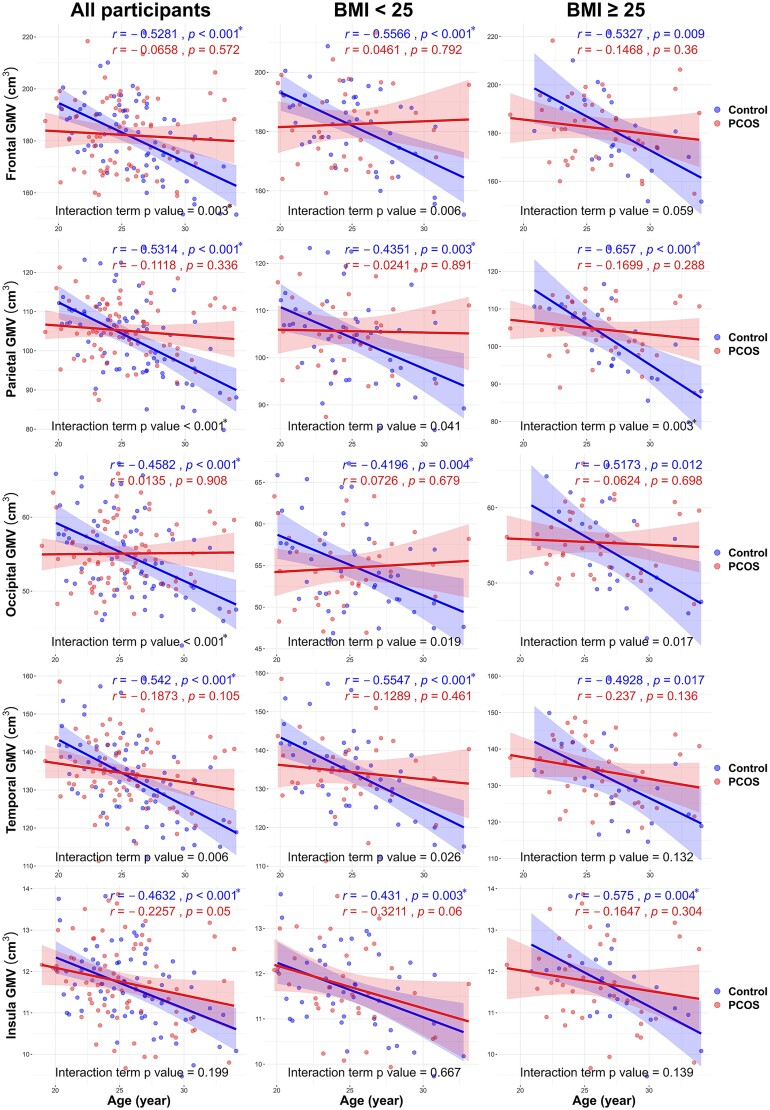
**Correlation of grey matter volume (GMV) with age of women with and without PCOS across the frontal, parietal, occipital, temporal and insula regions of the brain.** The correlation of GMV with age in other brain regions is shown in Supplementary Fig. 2. Pearson’s correlation coefficient (*r*) is reported, and the interaction term refers to the interaction between age and regional GMV in linear regression (control group: *n* = 68, PCOS group: *n* = 76). Bold lines represent the trend lines of age and regional GMV, with shaded areas indicating 95% confidence intervals. *P < 0.005 was considered statistically significant according to Bonferroni’s correction for multiple comparisons.

The association between regional grey matter volume and age in specific brain regions is illustrated in Fig. 2 and Supplementary Fig. 2. In women without PCOS, regardless of obesity, grey matter volume significantly decreased with age in the frontal, parietal, occipital, temporal, insula and limbic regions. Conversely, in women with PCOS, increasing age did not significantly correlate with a decrease in grey matter volume across all brain regions, regardless of obesity. The decline in grey matter volume with age differed significantly between women with and without PCOS in the frontal, parietal and occipital regions.

**Figure 3:**
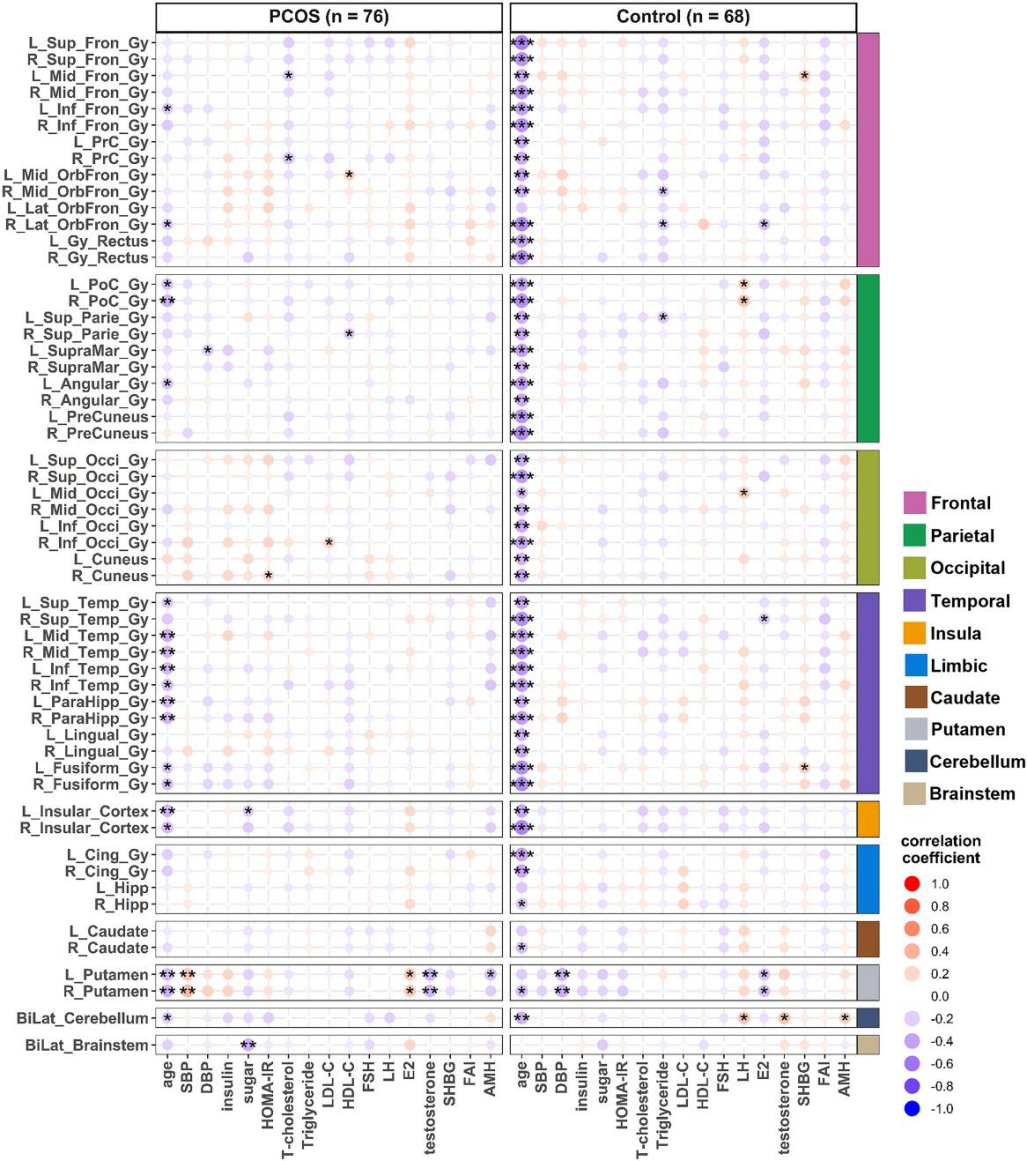
**Heatmap of grey matter volume of 56 specific brain regions plotted against age and other 18 metabolic and hormonal variables in women with and without PCOS.** BMI and DHEAS adjusted Pearson’s correlation coefficient between grey matter volume and age, blood pressure levels, circulating metabolic and hormonal variables as indicated. SBP, systolic blood pressure; DBP, diastolic blood pressure; LDL-C, low-density lipoprotein cholesterol; HDL-C, high-density lipoprotein cholesterol; FSH, follicle-stimulating hormone; LH, luteinizing hormone; E2, oestradiol; DHEAS, dehydroepiandrosterone sulphate; SHBG, sex hormone-binding globulin; FAI, free androgen index; AMH, anti-Müllerian hormone. The complete names of the abbreviations for all brain regions of interest (ROIs) are listed in the supplementary file titled Glossary_Complete_names_of_brain_ROIs. ***P < 0.001, **P < 0.01, *P < 0.05 (the *P*-values reported here were uncorrected for multiple comparisons due to exploratory analysis).

After adjusting for BMI and DHEAS levels, the associations between increasing age and a decrease in grey matter volume in women with PCOS, particularly in the frontal, parietal, temporal, insula and putamen regions, are shown in Fig. 3. The grey matter volume of specific gyri in each brain region and their associations with age, BMI and all the other metabolic and hormonal variables without adjustments are shown in Supplementary Fig. 3.

### Cortical thickness

#### Global cortical thickness

The mean global cortical thickness showed no significant difference between women with and without PCOS or between obese and non-obese women (Table 2). Similar to the findings for grey matter volume, increasing age was significantly associated with a decrease in global cortical thickness in women without PCOS, but not in women with PCOS, regardless of obesity (Fig. 1B). By using univariate analysis to investigate the association between global cortical thickness and other variables including anthropometric measurements, hormone levels and metabolic profiles, the results in Table 3 showed that insulin levels, HOMA-IR and LDL-C revealed significant positive association with the global cortical thickness in all enrolled subjects. Similarly, the results of stepwise regression model for the global cortical thickness showed that only age, DHEAS and LDL-C remained in the final model for women with PCOS. In contrast, age, DHEAS and HOMA-IR remained in the final model for women without PCOS (Table 3). These findings suggest that DHEAS and insulin resistance are involved in the age-related loss of global cortical thickness in young women.

#### Regional cortical thickness

The average cortical thickness within specific brain regions and their corresponding gyri is given in Table 2 and Supplementary Table 4. The cortical thickness of the right superior parietal and right supramarginal gyri in the parietal region, as well as the right middle temporal gyrus in the temporal region, was significantly higher in women with PCOS compared with those without. In addition, the cortical thickness of the left posterior cingulate gyrus was significantly higher in non-obese women with PCOS (Supplementary Table 4).

**Figure 4:**
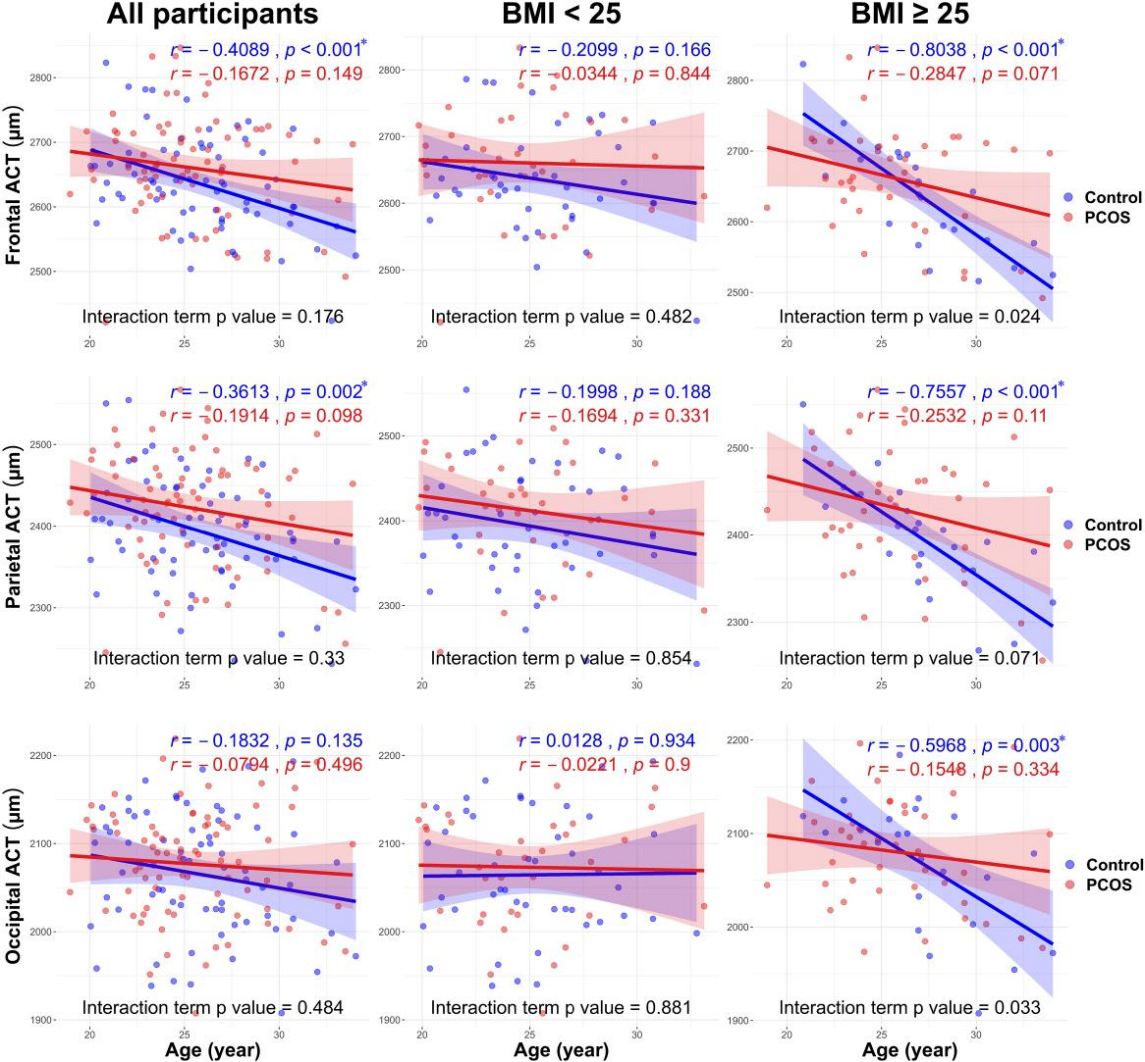
**Correlation of average cortical thickness (ACT) with age of women with and without PCOS across the frontal parietal and occipital regions of the brain.** The correlation of ACT with age in other brain regions is shown in Supplementary Fig. 4. Pearson’s correlation coefficient (*r*) is reported, and the interaction term refers to the interaction between age and regional ACT in linear regression (control group: *n* = 68, PCOS group: *n* = 76). Bold lines represent the trend lines of age and regional average cortical thickness, with shaded areas indicating 95% confidence intervals. *P < 0.008 was considered statistically significant according to Bonferroni’s correction for multiple comparisons.

**Figure 5:**
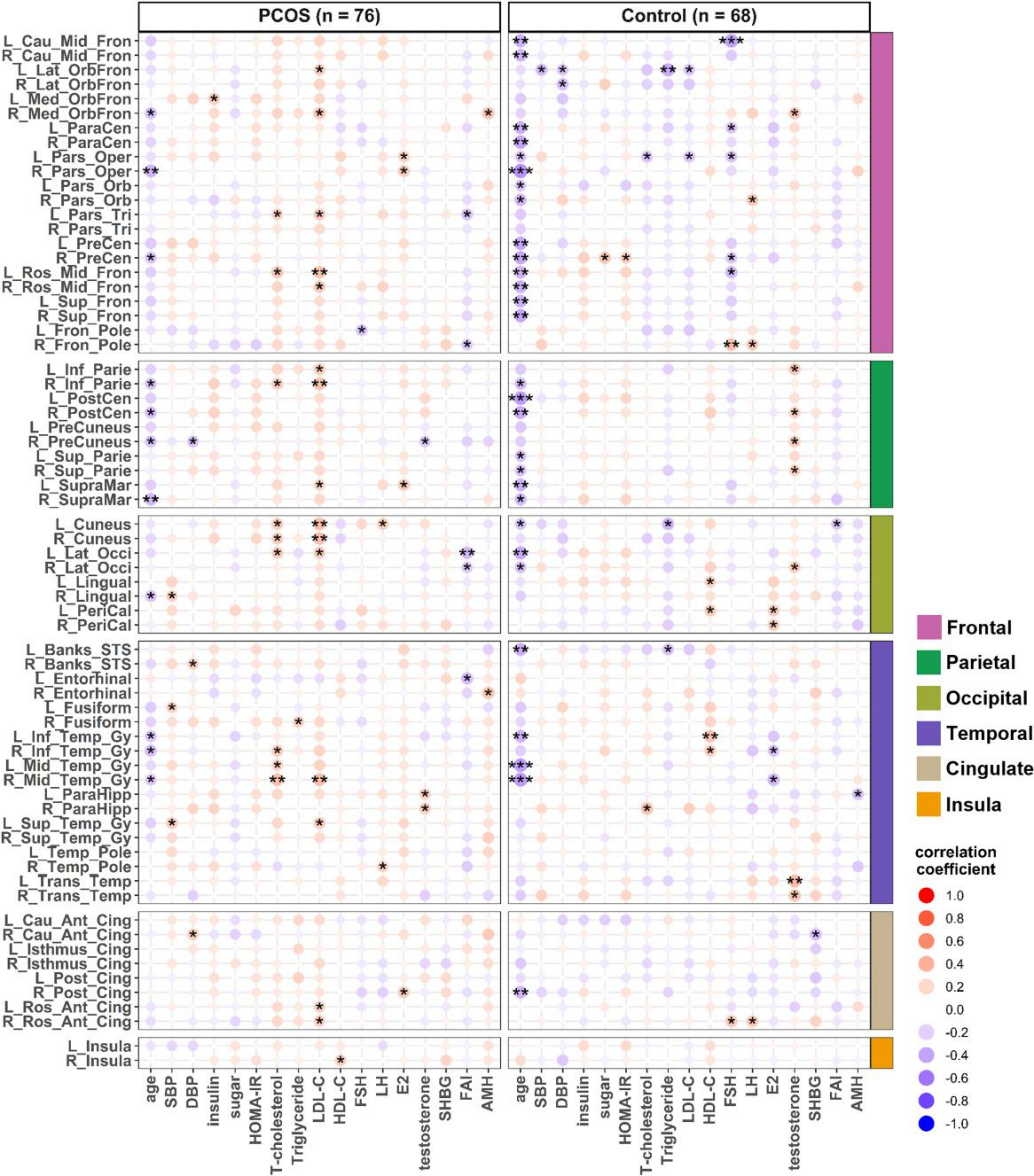
**Heatmap of cortical thickness of 68 specific brain regions plotted against age and other 18 metabolic and hormonal variables in women with and without PCOS.** BMI and DHEAS adjusted Pearson’s correlation coefficient between cortical thickness and age, blood pressure levels, circulating metabolic and hormonal variables as indicated. SBP, systolic blood pressure; DBP, diastolic blood pressure; LDL-C, low-density lipoprotein cholesterol; HDL-C, high-density lipoprotein cholesterol; FSH, follicle-stimulating hormone; LH, luteinizing hormone; E2, oestradiol; DHEAS, dehydroepiandrosterone sulphate; SHBG, sex hormone-binding globulin; FAI, free androgen index; AMH, anti-Müllerian hormone. The complete names of the abbreviations for all brain ROIs are listed in the supplementary file titled Glossary_Complete_names_of_brain_ROIs. ***P < 0.001, **P < 0.01, *P < 0.05 (the *P*-values reported here were uncorrected for multiple comparison due to exploratory analysis).

The relationship between regional cortical thickness and age in specific brain regions is illustrated in Fig. 4 and Supplementary Fig. 4. The cortical thickness of the frontal and parietal regions significantly decreased with age in women without PCOS, particularly among obese women. In contrast, a decrease in cortical thickness with age in the occipital and cingulate regions was only evident in the obese group. The negative association between age and regional cortical thickness in women with PCOS was not significant. However, after adjusting for BMI and DHEAS levels, increasing age became significantly associated with a decrease in regional cortical thickness in women with PCOS, especially in the frontal, parietal and temporal regions (Fig. 5 and Supplementary Fig. 5).

## Discussion

In this study, we observed an age-related decline in grey matter volume among women aged 20–35 years, regardless of their obesity status. However, when comparing women with PCOS to those without PCOS, the decline in total grey matter volume with age was significantly reduced in women with PCOS. The difference was particularly significant in some specific brain regions including the frontal, parietal, occipital, temporal and limbic regions. These areas are known to be rich in androgen receptors and susceptible to atrophy or structural changes in conditions such as Alzheimer’s disease and emotional disorders, or in the presence of sex differences.^41-43^ Women with and without PCOS have comparable global grey matter volumes in the younger age groups (Supplementary Fig. 6). The reduction in cortical thickness with age was more pronounced in women without PCOS, while the difference in age-related cortical thickness decline between women with and without PCOS did not reach statistical significance. In addition, the impact of obesity on cortical thickness loss was notable, representing a more pronounced decline with age in obese women, irrespective of the presence of PCOS. This suggests that obesity may exacerbate the process of brain ageing, particularly in regions susceptible to atrophy and structural changes. The negative association between age and both grey matter volume and cortical thickness became even more pronounced after adjusting for DHEAS levels. This highlights the potential role of androgens in influencing age-related changes in brain structure. Our findings suggest that hyperandrogenaemia and obesity may contribute to structural changes in the brain with age in young women with PCOS.

Androgens have been reported to have neuroprotective properties, influence brain plasticity and potentially mitigate the progression of Alzheimer’s disease.^20,22^ Early deprivation of oestrogens and androgens is associated with the onset of Alzheimer’s disease, while androgen administration may help reduce inflammation and slow the progression of neurodegenerative disorders.^44^ Women with PCOS are characterized by hyperandrogenaemia including elevated adrenal and ovarian androgens. The primary androgen secreted by the ovaries is testosterone, while DHEAS is mainly produced by the adrenal glands. Although levels of both DHEAS and testosterone decline with age, they can be converted into each other and have exerted different effects on metabolism. DHEAS can naturally convert to testosterone and subsequently to oestrogen under normal physiological conditions, exerting its effects through both androgen and oestrogen receptors.^45^ In contrast to testosterone, higher circulating levels of DHEAS are generally associated with more favourable metabolic parameters such as lower body weight, reduced abdominal adiposity, an improved metabolic profile, higher insulin sensitivity and reduced vascular dysfunction.^27,46^ A decrease in the DHEA/cortisol ratio with age has been reported to be associated with the progression of atherosclerosis and the impairment of cognitive and affective performance.^47^ DHEA supplementation has been reported to have benefits in preventing age-related conditions such as depression, cognitive disorders, obesity, diabetes, cancer and heart disease and may help counteract the age-related decline in sexual function and bone mass.^48,49^ In this study, the slower decline rate of global grey matter volume and cortical thickness with age in women with PCOS may be related to their high DHEAS levels.

Similar to the role of DHEAS/DHEA, several factors involved in promoting cell growth and development and regulating energy homeostasis—such as insulin, growth hormone (GH), IGF-1 and leptin—have also been reported to exhibit neuroprotective effects and play a role in mitigating brain ageing.^50,51^ The decline in these factors with age has been associated with structural changes in the brain. Women with PCOS, adolescents and women with high lean body mass tend to have higher levels of DHEAS, IGF-1, leptin, insulin and GH, all of which may influence brain structure and the rate of brain atrophy.^20-22,50,51^ However, individuals with high BMI often have elevated adiposity and glucose levels, which is associated with low-grade inflammation. This inflammation is potentially harmful to both body cells and brain function and has been linked to accelerate brain ageing.^10,11^ Unfortunately, in this study, data on GH, IGF-1, cytokines, leptin and haemoglobin A1c (HbA1c) were not available, limiting our ability to differentiate the effects of obesity and DHEAS on brain structures independent of these potential confounding factors. It is also important to note that the levels of these hormones generally decline with age.

The pathogenesis of PCOS in women is linked to the interplay between abdominal obesity and hyperandrogenaemia.^19^ Either extreme obesity or hyperandrogenaemia could contribute to the development of PCOS. Previous studies have reported that total brain volume and grey matter volume were smaller in obese women with PCOS compared with obese women without PCOS, while such differences were not observed in lean women with and without PCOS.^6,52^ Another study, with a limited sample size and no control for obesity, reported no significant difference in brain volume between women with and without PCOS.^7^ The discrepancies in these findings might be due to insufficient sample size and not considering the effect of androgens in women with PCOS.

Total grey matter volume and cortical thickness are known to peak before the age of 6 years, after which they gradually decrease with age.^8^ The rate and extent of this age-related brain atrophy can be influenced by many factors such as genetics, lifestyle factors and the presence of certain medical conditions, for example, Type 2 diabetes, obesity, schizophrenia, bipolar disorders, etc.^9,12-14^ Women with PCOS are characterized by higher androgen levels, chronic anovulation and a higher prevalence of obesity and metabolic disturbances, including Type 2 diabetes, hyperlipidaemia and hypertension, which are risk factors for neurodegenerative disorders such as Alzheimer’s disease and brain atrophy.^15,16^ However, it is unclear whether brain ageing occurs more rapidly in women with PCOS compared with those without PCOS.^16,18^ A recent study reported that women with PCOS may experience poorer brain health by midlife.^53^ However, due to the cross-sectional design of this study, it cannot fully capture the true decline in grey matter volume and cortical thickness with age, which a longitudinal study would better address. Additionally, the study only included women aged 20–35 years, so the findings can apply to younger populations but may not be generalized to older women. Our study noted that while DHEAS levels are higher in women with PCOS than those without PCOS before age 35 years, the rate of DHEAS decline is faster in women with PCOS. This suggests that the findings for women over 35 years may differ. Therefore, although our study found a distinctive pattern of brain ageing in women with PCOS, emphasizing the roles of hyperandrogenaemia and obesity, these findings may not apply to older populations.

Male and female brains differ in structure and function.^43^ These differences carry implications for various neurodegenerative, emotional and psychiatric conditions. Many brain-related illnesses exhibit sex differences, potentially influenced by factors such as chromosomes and sex steroids such as androgens and oestrogens. Notably, women tend to have a higher prevalence of Alzheimer’s disease and certain emotional disorders compared with men. Additional evidence supporting the impact of androgens on brain ageing in women includes the reported effect on female brain development, potentially aligning its structure or function more closely with that of males by regulating microglial cell phagocytosis.^54^ Our study revealed higher grey matter volume in the putamen and postcentral gyrus in the parietal lobe, as well as greater average cortical thickness in various regions of the parietal lobe in women with PCOS compared with those without. These findings align with reported sex dimorphisms in brain morphology, where men typically have a larger putamen and experience a milder age-related decline in the parietal lobe compared with women.^55,56^

Future research should explore the risk of neurodegenerative disorders in older women with PCOS and unravel causal relationships between androgens, brain ageing and their interaction with obesity. In addition, the identification of markers for predicting the risk of dementia and potential benefits of hormone replacement, DHEAS and insulin sensitizer treatment, as well as weight managing programmes for women at risk might be necessary for further investigation. The function of specific brain regions that are more susceptible to obesity and androgens also requires further evaluation in women with PCOS in future studies.

This study, while being the largest cross-sectional case–control study to date investigating the brain structure in women with PCOS using MRI, has several limitations. It is a single-site study with a relatively modest sample size and a cross-sectional design, which limits the ability to track longitudinal changes over time. Furthermore, the homogenous ancestry of the participants may restrict the generalizability of the findings to more diverse populations. The lack of replication through interventional studies, such as those involving androgen blockers, or through animal models, also limits the ability to establish causal relationships. Finally, while our findings suggest that young women with PCOS exhibit a reduced age-related decline in grey matter volume and cortical thickness, with DHEAS potentially playing a key role, women with PCOS still remain at risk for brain ageing. Therefore, future longitudinal studies and functional assessments of cognitive and emotional disorders, as well as their relationships with brain structures, DHEAS levels and obesity, are needed to validate and expand upon these findings.

Women with PCOS exhibit a distinctive pattern of brain ageing, marked by a reduced age-related decline in grey matter volume and cortical thickness, with DHEAS levels playing a significant role. The study provides valuable information for future strategies addressing brain ageing in high-risk populations.

## Supplementary Material

fcaf052_Supplementary_Data

## Data Availability

The data supporting the findings of this study are available within the article and its Supplementary material. Raw data including respective brain MRI images are not publicly available due to the privacy of research participants, but the secondary data will be made available from the corresponding authors upon request. The codes adopted for statistical analysis in this study are also available in the Supplementary material.
