# Supplementary material for: Influence of dehydroepiandrosterone sulphate levels on the slower age-related decline in grey matter in younger women with polycystic ovary syndrome
Source: Brain Commun. 2025 Feb 5;7(1):fcaf052. doi: 10.1093/braincomms/fcaf052 (PMC11829216; doi:10.1093/braincomms/fcaf052)
Supplement: fcaf052_Supplementary_Data [file fcaf052_supplementary_data.zip › Supplementary_Figures.pdf]

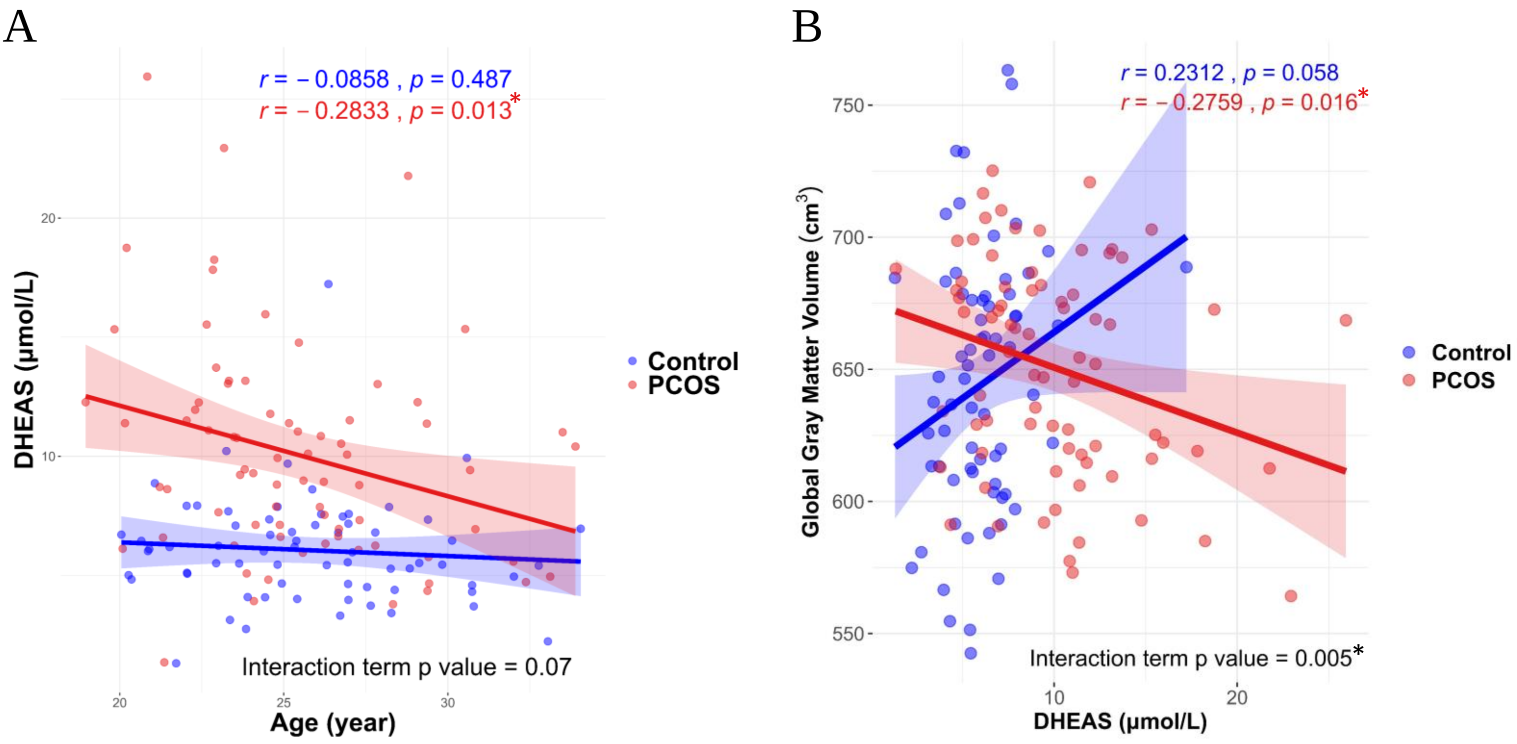

Supplementary Figure 1. The Age-Related Changes in DHEAS levels and it's Association with Global Gray Matter Volume in Women with and without PCOS.

(A) Illustrates the age-related changes in DHEAS levels with age between 20-35 y/o in women with and without PCOS. Notably, a significant decline in DHEAS levels is observed with increasing age in women with PCOS, while such a decline is not apparent in those without PCOS. **(B)**. Demonstrates the association between global gray matter volume and DHEAS levels in women with and without PCOS. Notably, global gray matter volume is positively correlated to the DHEAS levels in women without PCOS, but negatively correlated to DHEAS levels in women with PCOS. The statistically significant interaction term indicating the divergence in global gray matter volume associated with DHEAS levels in women with and without PCOS. *r* is the Pearson correlation coefficient; the interaction term is the interaction between DHEAS levels and global gray matter volume or age in linear regression (Control group: N= 68, PCOS group: N=76). Bold lines represent the trend lines, while the shaded areas indicate the 95% confidence intervals.
* $P < 0.05$ was considered statistically significant.

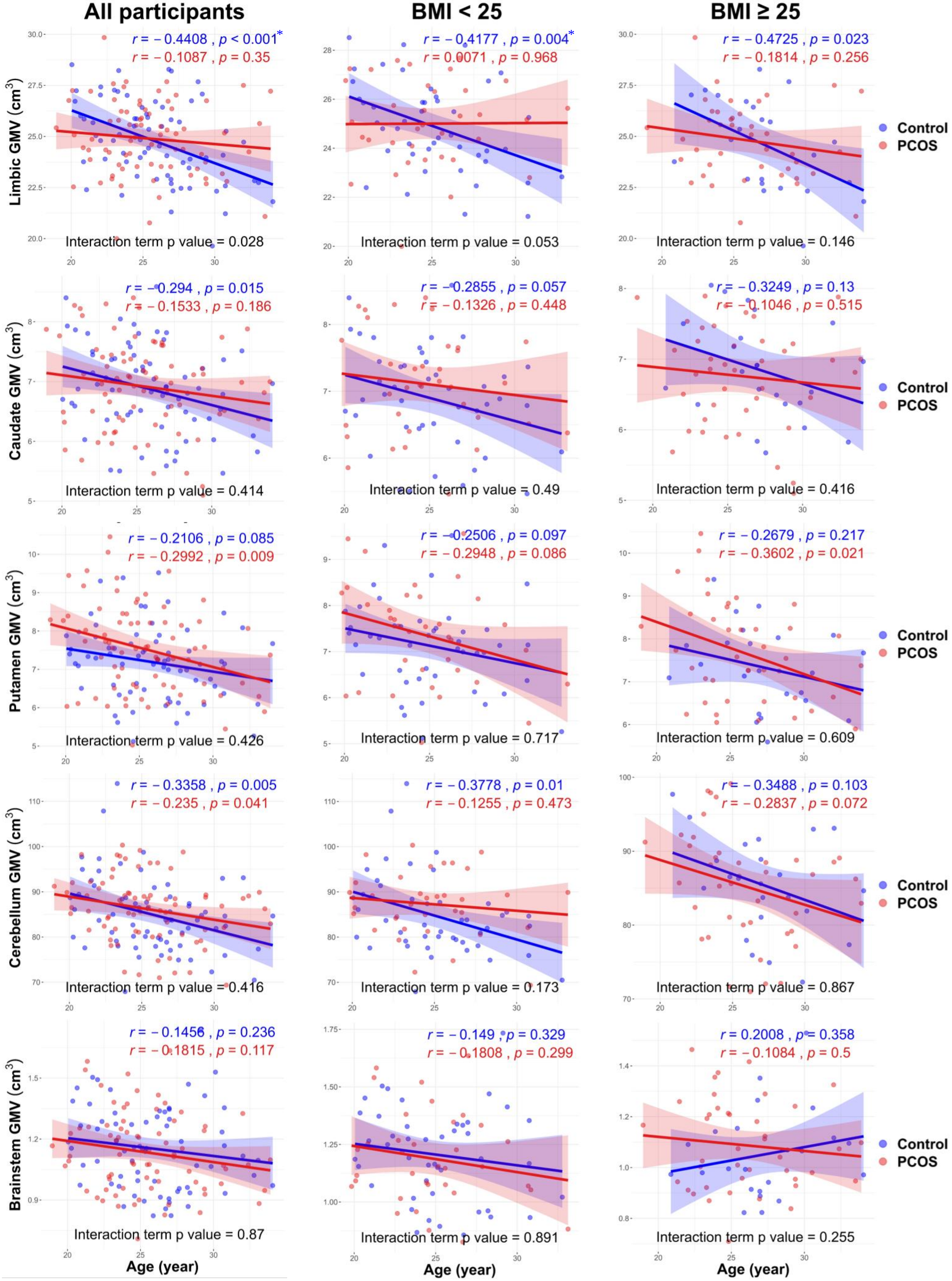

Supplementary Figure 2. Correlation of gray matter volume (GMV) with age of women with and without PCOS across the limbic, caudate, putamen, cerebellum and brain stem regions of the brain. Pearson correlation coefficient (*r*) is reported and the interaction term refers to the interaction between age and regional GMV in linear regression (Control group: N= 68, PCOS group: N=76). Bold lines represent the trend lines of age and regional GMV, with shaded areas indicating 95% confidence intervals. *$P < 0.005$ was considered statistically significant according to Bonferroni correction for multiple comparison.

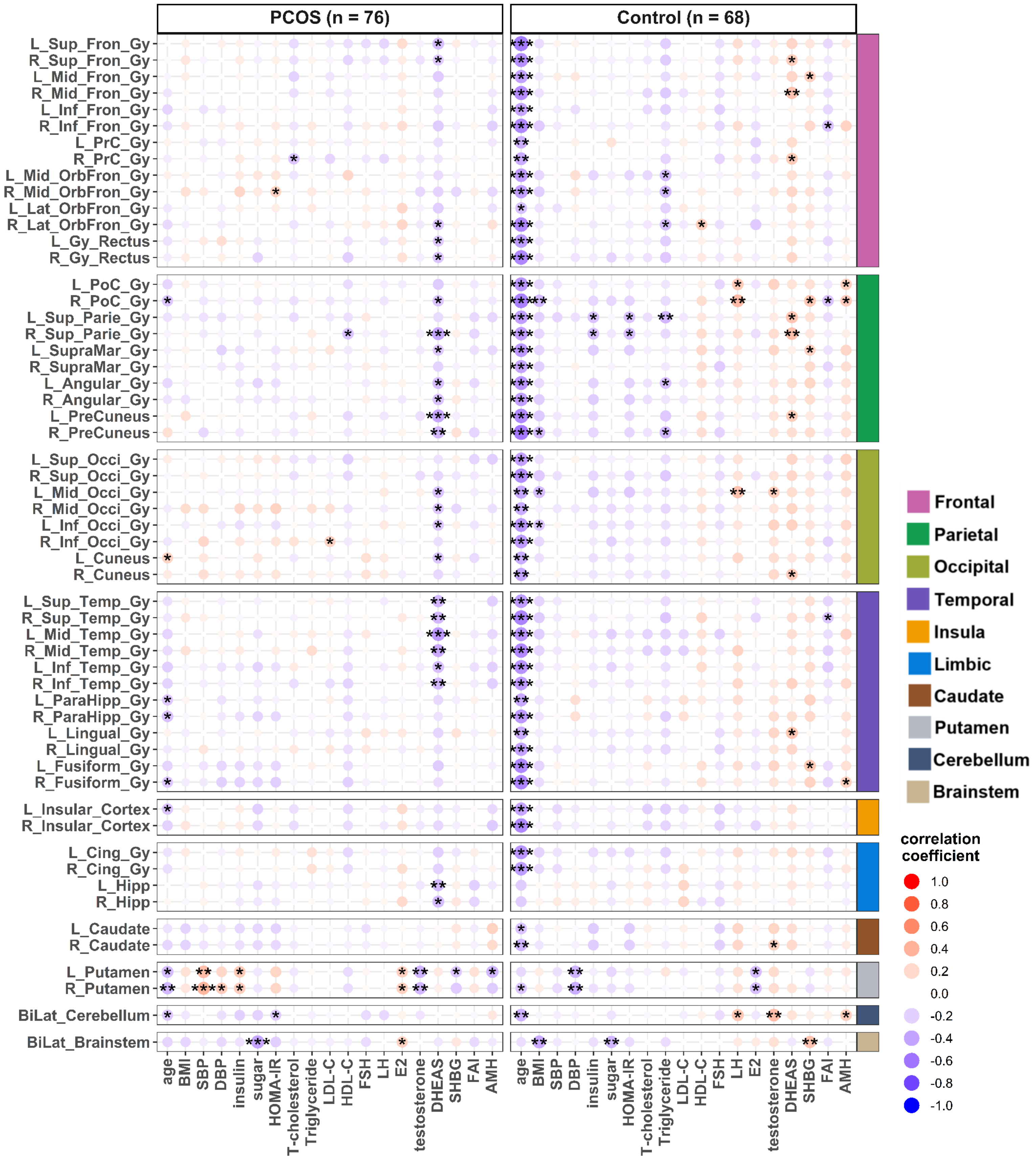

Supplementary Figure 3. Heatmap of gray matter volume of 56 specific brain region plotted against age and other 18 metabolic and hormonal variables in women with and without PCOS. Pearson correlation coefficient between gray matter volume and age, BMI, blood pressure levels, circulating metabolic and hormonal variables as indicated. SBP: systolic blood pressure; DBP: diastolic blood pressure; LDL-C: low density lipoprotein cholesterol; HDL-C: high density lipoprotein cholesterol; FSH: follicle stimulating hormone; LH: luteinizing hormone; E2: estradiol; DHEAS: dehydroepiandrosterone sulfate; SHBG: sex hormone binding globulin; FAI: free androgen index; AMH: anti-Müllerian hormone. The complete names of the abbreviations for all brain regions of interest (ROIs) are listed in the supplementary file. ***P < 0.001, **P < 0.01, *P < 0.05. (The P values reported here were uncorrected for multiple comparison due to exploratory analysis)

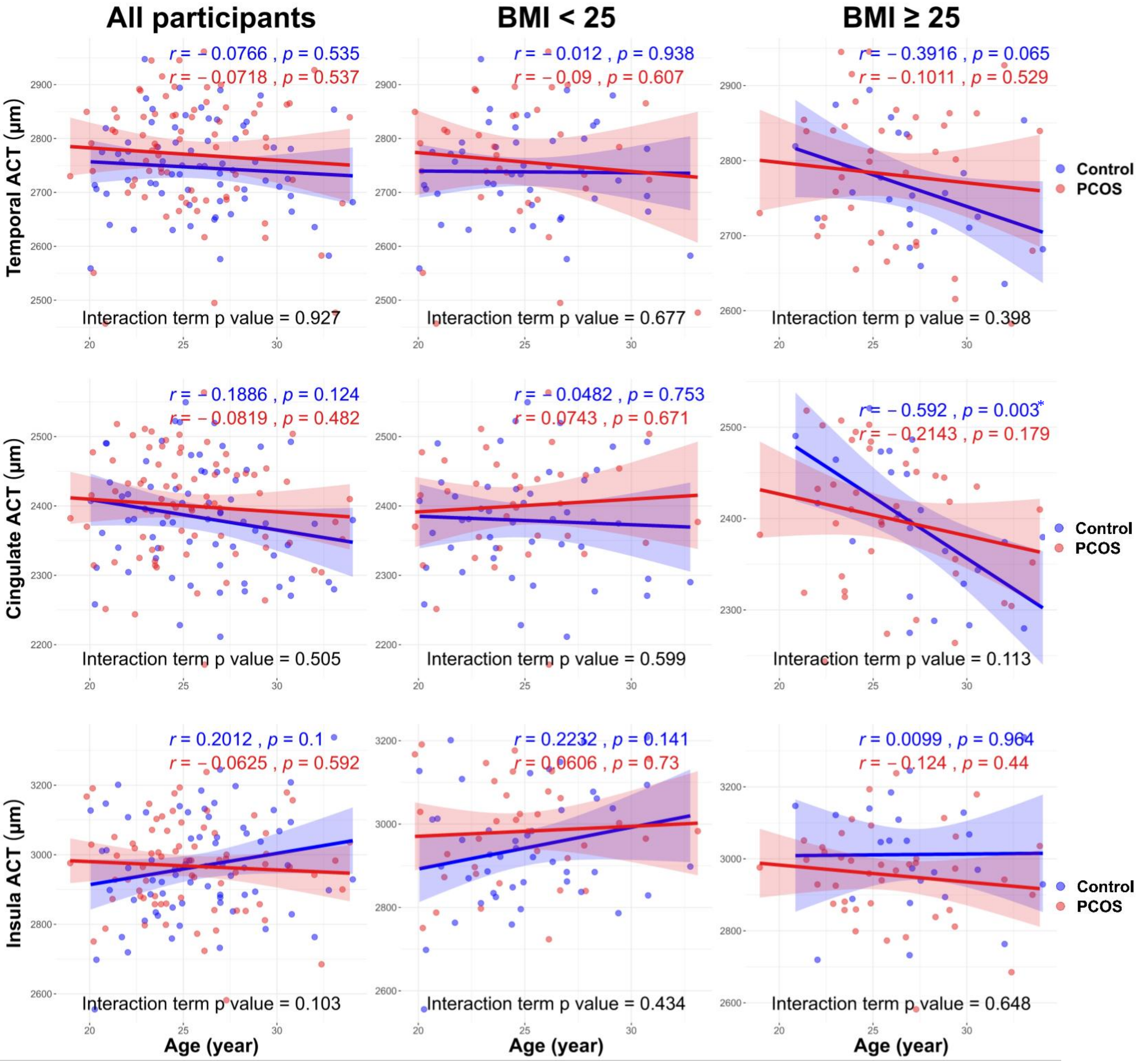

Supplementary Figure 4. Correlation of average cortical thickness (ACT) with age of women with and without PCOS across the temporal, cingulate, and insula regions of the brain. Pearson correlation coefficient (*r*) is reported and the interaction term refers to the interaction between age and regional ACT in linear regression (Control group: N= 68, PCOS group: N=76). Bold lines represent the trend lines of age and regional ACT, with shaded areas indicating 95% confidence intervals. **P* < 0.008 was considered statistically significant according to Bonferroni correction for multiple comparison.

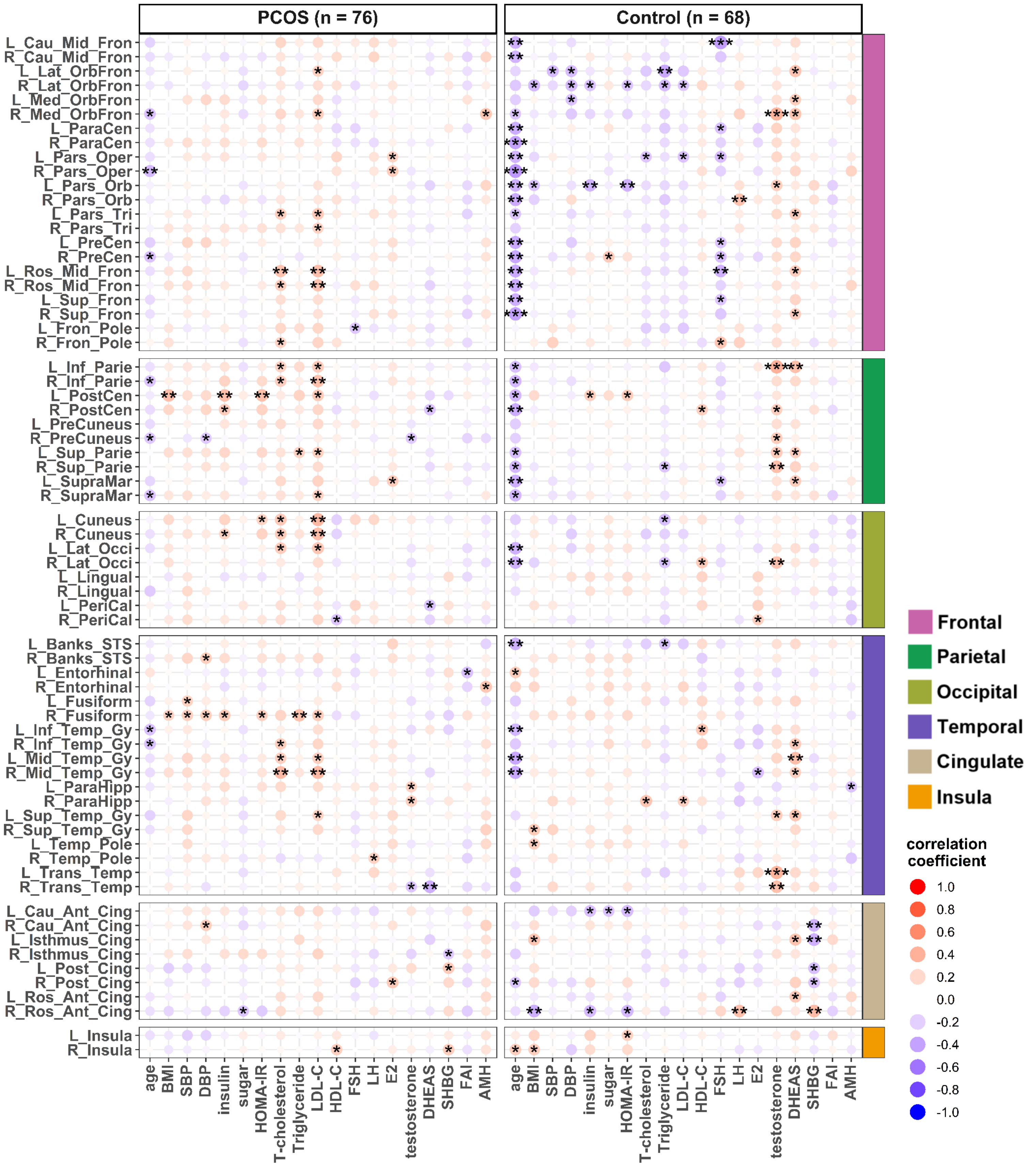

Supplementary Figure 5. Heatmap of cortical thickness of 68 specific brain region plotted against age and other 18 metabolic and hormonal variables in women with and without PCOS. Pearson correlation coefficient between cortical thickness and age, BMI, blood pressure levels, circulating metabolic and hormonal variables as indicated. SBP: systolic blood pressure; DBP: diastolic blood pressure; LDL-C: low density lipoprotein cholesterol; HDL-C: high density lipoprotein cholesterol; FSH: follicle stimulating hormone; LH: luteinizing hormone; E2: estradiol; DHEAS: dehydroepiandrosterone sulfate; SHBG: sex hormone binding globulin; FAI: free androgen index; AMH: anti-Müllerian hormone. The complete names of the abbreviations for all brain regions of interest (ROIs) are listed in the supplementary file. ***P < 0.001, **P < 0.01, *P < 0.05. (The P values reported here were uncorrected for multiple comparison due to exploratory analysis).

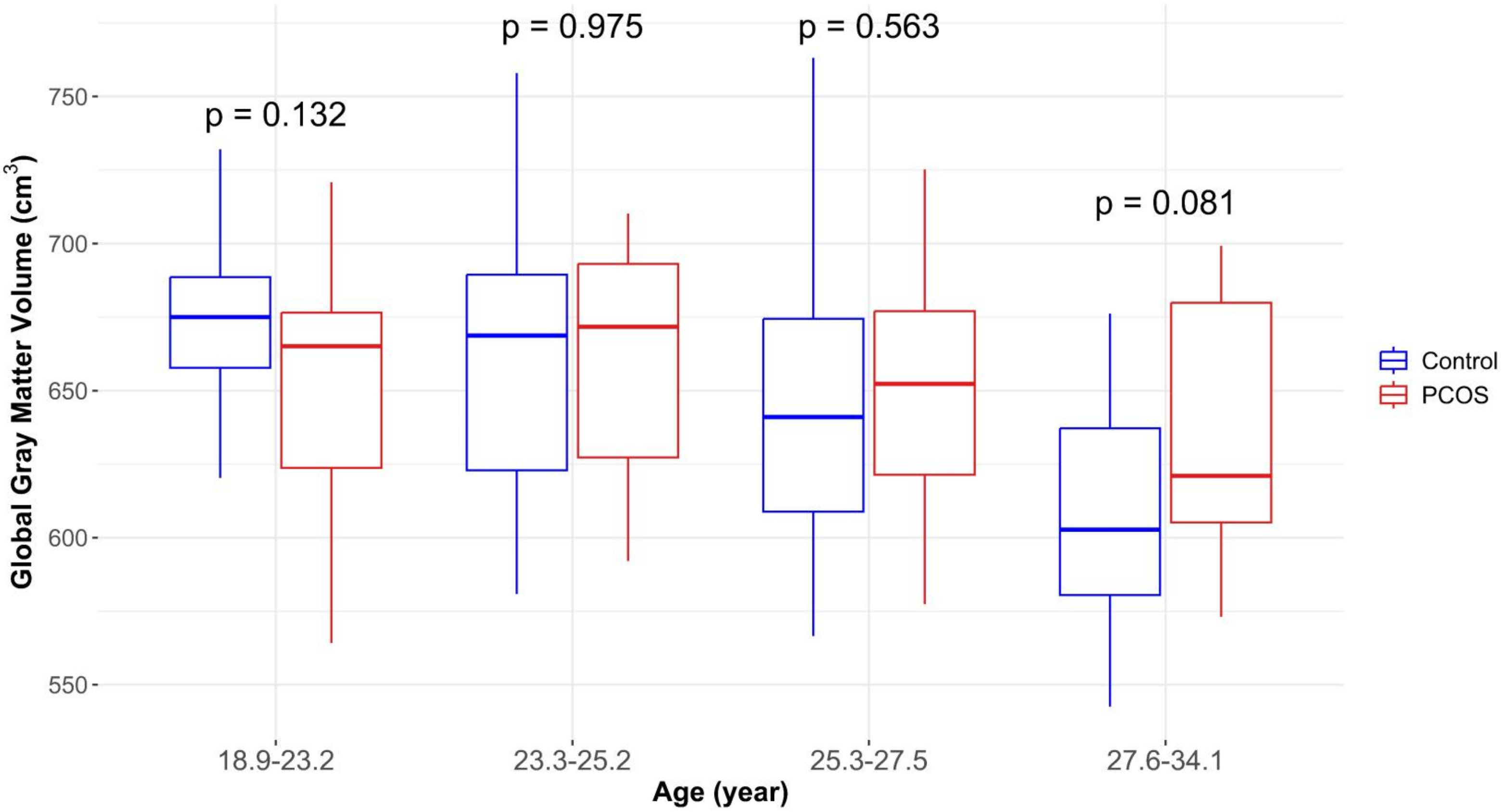

Supplementary Figure 6. Comparison of global gray matter volume in four age groups, from the youngest to the oldest, based on age quartiles, between women with and without PCOS. The number of control individuals in each group was 16, 15, 18, and 19, respectively, while the number of PCOS individuals was 20, 21, 18, and 17, respectively. The results show no statistically significant differences in global gray matter volume between the control and PCOS groups across the four age groups, with *P* values of 0.132, 0.975, 0.563, and 0.081 (nonparametric method: Wilcoxon rank-sum test). According to the data, there is no evidence to show that the PCOS group had a lower gray matter volume to begin with.
