# Supplementary material for: Influence of dehydroepiandrosterone sulphate levels on the slower age-related decline in grey matter in younger women with polycystic ovary syndrome
Source: Brain Commun. 2025 Feb 5;7(1):fcaf052. doi: 10.1093/braincomms/fcaf052 (PMC11829216; doi:10.1093/braincomms/fcaf052)
Supplement: fcaf052_Supplementary_Data [file fcaf052_supplementary_data.zip › Supplementary_material_Glossary_Complete_names_of_brain_ROIs.docx]

**Gray matter volume**

**Code - ROI Name**

**Frontal**

L_Sup_Fron_Gy - Left superior frontal gyrus

R_Sup_Fron_Gy - Right superior frontal gyrus

L_Mid_Fron_Gy - Left middle frontal gyrus

R_Mid_Fron_Gy - Right middle frontal gyrus

L_Inf_Fron_Gy - Left inferior frontal gyrus

R_Inf_Fron_Gy - Right inferior frontal gyrus

L_PrC_Gy - Left precentral gyrus

R_PrC_Gy - Right precentral gyrus

L_Mid_OrbFron_Gy - Left middle orbitofrontal gyrus

R_Mid_OrbFron_Gy - Right middle orbitofrontal gyrus

L_Lat_OrbFron_Gy - Left lateral orbitofrontal gyrus

R_Lat_OrbFron_Gy - Right lateral orbitofrontal gyrus

L_Gy_Rectus - Left gyrus rectus

R_Gy_Rectus - Right gyrus rectus

**Parietal**

L_PoC_Gy - Left postcentral gyrus

R_PoC_Gy - Right postcentral gyrus

L_Sup_Parie_Gy - Left superior parietal gyrus

R_Sup_Parie_Gy - Right superior parietal gyrus

L_SupraMar_Gy - Left supramarginal gyrus

R_SupraMar_Gy - Right supramarginal gyrus

L_Angular_Gy - Left angular gyrus

R_Angular_Gy - Right angular gyrus

L_PreCuneus - Left precuneus

R_PreCuneus - Right precuneus

**Occipital**

L_Sup_Occi_Gy - Left superior occipital gyrus

R_Sup_Occi_Gy - Right superior occipital gyrus

L_Mid_Occi_Gy - Left middle occipital gyrus

R_Mid_Occi_Gy - Right middle occipital gyrus

L_Inf_Occi_Gy - Left inferior occipital gyrus

R_Inf_Occi_Gy - Right inferior occipital gyrus

L_Cuneus - Left cuneus

R_Cuneus - Right cuneus

**Temporal**

L_Sup_Temp_Gy - Left superior temporal gyrus

R_Sup_Temp_Gy - Right superior temporal gyrus

L_Mid_Temp_Gy - Left middle temporal gyrus

R_Mid_Temp_Gy - Right middle temporal gyrus

L_Inf_Temp_Gy - Left inferior temporal gyrus

R_Inf_Temp_Gy - Right inferior temporal gyrus

L_ParaHipp_Gy - Left parahippocampal gyrus

R_ParaHipp_Gy - Right parahippocampal gyrus

L_Lingual_Gy - Left lingual gyrus

R_Lingual_Gy - Right lingual gyrus

L_Fusiform_Gy - Left fusiform gyrus

R_Fusiform_Gy - Right fusiform gyrus

**Insula**

L_Insular_Cortex - Left insular cortex

R_Insular_Cortex - Right insular cortex

**Limbic**

L_Cing_Gy - Left cingulate gyrus

R_Cing_Gy - Right cingulate gyrus

L_Hipp - Left hippocampus

R_Hipp - Right hippocampus

**Caudate**

L_Caudate - Left caudate

R_Caudate - Right caudate

**Putamen**

L_Putamen - Left putamen

R_Putamen - Right putamen

**Cerebellum**

BiLat_Cerebellum - bilateral cerebellum

**Brainstem**

BiLat_Brainstem - bilateral brainstem

**Cortical thickness**

**Code - ROI Name**

**Frontal**

L_Cau_Mid_Fron - Left caudal middle frontal

R_Cau_Mid_Fron - Right caudal middle frontal

L_Lat_OrbFron - Left lateral orbitofrontal

R_Lat_OrbFron - Right lateral orbitofrontal

L_Med_OrbFron - Left medial orbitofrontal

R_Med_OrbFron - Right medial orbitofrontal

L_ParaCen - Left paracentral

R_ParaCen - Right paracentral

L_Pars_Oper - Left pars opercularis

R_Pars_Oper - Right pars opercularis

L_Pars_Orb - Left pars orbitalis

R_Pars_Orb - Right pars orbitalis

L_Pars_Tri - Left pars triangularis

R_Pars_Tri - Right pars triangularis

L_PreCen - Left precentral

R_PreCen - Right precentral

L_Ros_Mid_Fron - Left rostral middle frontal

R_Ros_Mid_Fron - Right rostral middle frontal

L_Sup_Fron - Left superior frontal

R_Sup_Fron - Right superior frontal

L_Fron_Pole - Left frontal pole

R_Fron_Pole - Right frontal pole

**Parietal**

L_Inf_Parie - Left inferior parietal

R_Inf_Parie - Right inferior parietal

L_PostCen - Left postcentral

R_PostCen - Right postcentral

L_PreCuneus - Left precuneus

R_PreCuneus - Right precuneus

L_Sup_Parie - Left superior parietal

R_Sup_Parie - Right superior parietal

L_SupraMar - Left supramarginal

R_SupraMar - Right supramarginal

**Occipital**

L_Cuneus - Left cuneus

R_Cuneus - Right cuneus

L_Lat_Occi - Left lateral occipital

R_Lat_Occi - Right lateral occipital

L_Lingual - Left lingual

R_Lingual - Right lingual

L_PeriCal - Left pericalcarine

R_PeriCal - Right pericalcarine

**Temporal**

L_Banks_STS - Left banks of the superior temporal sulcus

R_Banks_STS - Right banks of the superior temporal sulcus

L_Entorhinal - Left entorhinal

R_Entorhinal - Right entorhinal

L_Fusiform - Left fusiform

R_Fusiform - Right fusiform

L_Inf_Temp_Gy - Left inferior temporal gyrus

R_Inf_Temp_Gy - Right inferior temporal gyrus

L_Mid_Temp_Gy - Left middle temporal gyrus

R_Mid_Temp_Gy - Right middle temporal gyrus

L_ParaHipp - Left parahippocampal

R_ParaHipp - Right parahippocampal

L_Sup_Temp_Gy - Left superior temporal gyrus

R_Sup_Temp_Gy - Right superior temporal gyrus

L_Temp_Pole - Left temporal pole

R_Temp_Pole - Right temporal pole

L_Trans_Temp - Left transverse temporal

R_Trans_Temp - Right transverse temporal

**Cingulate**

L_Cau_Ant_Cing - Left caudal anterior cingulate

R_Cau_Ant_Cing - Right caudal anterior cingulate

L_Isthmus_Cing - Left isthmus cingulate

R_Isthmus_Cing - Right isthmus cingulate

L_Post_Cing - Left posterior cingulate

R_Post_Cing - Right posterior cingulate

L_Ros_Ant_Cing - Left rostral anterior cingulate

R_Ros_Ant_Cing - Right rostral anterior cingulate

**Insula**

L_Insula - Left insula

R_Insula - Right insula
