# Supplementary material for: Influence of dehydroepiandrosterone sulphate levels on the slower age-related decline in grey matter in younger women with polycystic ovary syndrome
Source: Brain Commun. 2025 Feb 5;7(1):fcaf052. doi: 10.1093/braincomms/fcaf052 (PMC11829216; doi:10.1093/braincomms/fcaf052)
Supplement: fcaf052_Supplementary_Data [file fcaf052_supplementary_data.zip › Supplementary_Tables.docx]

**Supplementary Table 1 Demographic and clinical characteristics of women with and without PCOS**

|  | PCOS | | | Control | | | Two-way ANOVA *P* value | | |
| --- | --- | --- | --- | --- | --- | --- | --- | --- | --- |
|  | All  (n=76) | BMI<25 kg/m^2^  (n=35) | BMI≥25 kg/m^2^  (n=41) | All  (n=68) | BMI<25 kg/m^2^  (n=45) | BMI≥25 kg/m^2^  (n=23) | Main effect of PCOS | Main effect of BMI | Interaction effect |
| FSH (mIU/mL) | 6.4 (2.0) | 6.1 (1.7) | 6.6 (2.1) | 7.1 (1.8) | 7.0 (1.8) | 7.3 (1.8) | **0.03*** | 0.226 | 0.799 |
| LH (mIU/mL) | 11.0 (6.0) | 12.2 (5.0) | 10.0 (6.6) | 4.4 (2.0) | 4.6 (2.1) | 4.0 (1.7) | **<0.001*** | 0.054 | 0.303 |
| E2 (pg/mL) | 79.1 (77.9) | 95.1 (95.6) | 65.5 (56.6) | 36.2 (17.2) | 39.4 (18.4) | 29.8 (12.4) | **<0.001*** | **0.037*** | 0.313 |

Data are presented as mean (standard deviation).

FSH: follicle stimulating hormone; LH: luteinizing hormone; E2: estradiol.

*****P<0.05 was considered statistically significant by two-way ANOVA test

**Supplementary Table 2 Univariate linear regression analysis all the variable contribute to global gray matter volume and global cortical thickness**

|  | Global GMV Total | Global GMV PCOS | Global GMV Control | Global CT Total | Global CT PCOS | Global CT Control |
| --- | --- | --- | --- | --- | --- | --- |
|  | Univariate | Univariate | Univariate | Univariate | Univariate | Univariate |
| FSH | -2.43 (1.94) | -0.48 (2.42) | -4.53 (3.26) | -4.87 (3.55) | -0.44 (4.94) | -8.78 (5.24) |
| LH | 0.32 (0.66) | -0.48 (0.79) | 4.79 (2.95) | 1.48 (1.21) | 0.90 (1.61) | -1.67 (4.86) |
| E2 | 0.05 (0.06) | 0.05 (0.06) | -0.29 (0.35) | 0.13 (0.11) | 0.10 (0.12) | -0.25 (0.57) |

Unit of gray matter volume: cm^3^; unit of cortical thickness: µm; Data are presented as Beta (standard error).

FSH: follicle stimulating hormone; LH: luteinizing hormone; E2: estradiol.

**Supplementary Table 3 Gray matter volume of different regions of brain**

|  | PCOS | | | | Control | | | | P value of PCOS v.s. Control | | |
| --- | --- | --- | --- | --- | --- | --- | --- | --- | --- | --- | --- |
|  | All  (n=76) | BMI<25 Kg/m^2^  (n=35) | BMI≥25 Kg/m^2^  (n=41) | P value of BMI< and ≥25 Kg/m^2^ in PCOS | All  (n=68) | BMI<25 Kg/m^2^  (n=45) | BMI≥25 Kg/m^2^  (n=23) | P value of BMI< and ≥25 Kg/m^2^ in Control | ALL | BMI<25 Kg/m^2^ | BMI≥25 Kg/m^2^ |
| **Frontal** | | | | | | | | | | | |
| L Sup Fron Gy | 31.8 (2.6) | 31.7 (2.7) | 31.8 (2.6) | 0.852 | 31.7 (2.8) | 31.8 (2.6) | 31.4 (3.1) | 0.599 | 0.853 | 0.850 | 0.602 |
| R Sup Fron Gy | 30.7 (2.6) | 30.6 (2.3) | 30.8 (2.9) | 0.683 | 30.8 (2.8) | 30.8 (2.6) | 30.7 (3.1) | 0.903 | 0.847 | 0.648 | 0.913 |
| L Mid Fron Gy | 23.1 (2.1) | 23.2 (2.1) | 23.0 (2.1) | 0.602 | 23.0 (2.1) | 23.1 (2.0) | 22.7 (2.4) | 0.455 | 0.699 | 0.791 | 0.604 |
| R Mid Fron Gy | 23.4 (2.1) | 23.5 (2.2) | 23.2 (2.1) | 0.573 | 23.3 (2.2) | 23.4 (1.9) | 23.2 (2.8) | 0.760 | 0.926 | 0.802 | 0.956 |
| L Inf Fron Gy | 12.0 (1.1) | 12.1 (1.2) | 11.9 (1.1) | 0.500 | 11.9 (1.1) | 11.9 (1.0) | 11.9 (1.3) | 0.978 | 0.611 | 0.445 | 0.954 |
| R Inf Fron Gy | 12.7 (1.1) | 12.7 (1.2) | 12.7 (1.1) | 0.758 | 12.6 (1.3) | 12.7 (1.1) | 12.3 (1.6) | 0.270 | 0.506 | 0.842 | 0.242 |
| L PrC Gy | 13.2 (1.1) | 13.2 (1.1) | 13.1 (1.1) | 0.876 | 12.9 (1.1) | 12.8 (1.0) | 13.2 (1.3) | 0.262 | 0.221 | 0.134 | 0.952 |
| R PrC Gy | 12.3 (1.1) | 12.4 (1.0) | 12.3 (1.2) | 0.627 | 12.3 (1.0) | 12.2 (1.0) | 12.4 (1.0) | 0.421 | 0.657 | 0.345 | 0.677 |
| L Mid OrbFron Gy | 5.4 (0.5) | 5.4 (0.4) | 5.4 (0.5) | 0.655 | 5.4 (0.5) | 5.4 (0.4) | 5.3 (0.7) | 0.697 | 0.676 | 0.902 | 0.552 |
| R Mid OrbFron Gy | 5.9 (0.6) | 5.8 (0.6) | 5.9 (0.6) | 0.356 | 5.8 (0.5) | 5.8 (0.5) | 5.7 (0.6) | 0.581 | 0.200 | 0.839 | 0.152 |
| L Lat OrbFron Gy | 3.7 (0.4) | 3.8 (0.4) | 3.6 (0.4) | 0.193 | 3.7 (0.4) | 3.7 (0.4) | 3.7 (0.4) | 0.589 | 0.933 | 0.662 | 0.837 |
| R Lat OrbFron Gy | 3.5 (0.3) | 3.5 (0.3) | 3.4 (0.4) | 0.423 | 3.4 (0.3) | 3.4 (0.3) | 3.4 (0.3) | 0.650 | 0.351 | 0.323 | 0.581 |
| L Gy Rectus | 2.2 (0.2) | 2.2 (0.2) | 2.2 (0.2) | 0.867 | 2.2 (0.2) | 2.2 (0.2) | 2.2 (0.3) | 0.944 | 0.670 | 0.792 | 0.799 |
| R Gy Rectus | 2.5 (0.2) | 2.5 (0.2) | 2.5 (0.2) | 0.807 | 2.5 (0.3) | 2.5 (0.3) | 2.5 (0.3) | 0.838 | 0.750 | 0.657 | 0.971 |
| **Parietal** | | | | | | | | | | | |
| L PoC Gy | 11.3 (0.9) | 11.3 (0.9) | 11.3 (0.9) | 0.713 | 10.9 (1.0) | 10.9 (1.0) | 10.8 (1.1) | 0.515 | **0.008*** | 0.051 | 0.063 |
| R PoC Gy | 10.4 (0.9) | 10.5 (0.9) | 10.3 (0.9) | 0.311 | 10.1 (0.9) | 10.3 (0.9) | 9.8 (0.9) | 0.070 | 0.047 | 0.184 | 0.040 |
| L Sup Parie Gy | 13.6 (1.2) | 13.7 (1.2) | 13.5 (1.2) | 0.405 | 13.3 (1.4) | 13.3 (1.3) | 13.1 (1.6) | 0.586 | 0.170 | 0.200 | 0.396 |
| R Sup Parie Gy | 13.4 (1.0) | 13.4 (1.1) | 13.4 (0.9) | 0.937 | 13.2 (1.4) | 13.3 (1.3) | 13.1 (1.6) | 0.526 | 0.450 | 0.763 | 0.408 |
| L SupraMar Gy | 9.0 (1.0) | 8.9 (1.0) | 9.0 (0.9) | 0.790 | 9.0 (1.2) | 9.0 (1.2) | 8.8 (1.2) | 0.531 | 0.904 | 0.760 | 0.544 |
| R SupraMar Gy | 8.6 (0.8) | 8.6 (0.9) | 8.7 (0.8) | 0.895 | 8.4 (1.0) | 8.5 (1.0) | 8.2 (1.1) | 0.346 | 0.147 | 0.539 | 0.124 |
| L Angular Gy | 11.3 (1.0) | 11.4 (1.0) | 11.2 (1.0) | 0.440 | 11.2 (1.5) | 11.3 (1.4) | 10.9 (1.6) | 0.243 | 0.539 | 0.795 | 0.337 |
| R Angular Gy | 12.3 (1.1) | 12.5 (1.2) | 12.2 (1.1) | 0.230 | 12.1 (1.4) | 12.2 (1.4) | 11.9 (1.5) | 0.360 | 0.403 | 0.409 | 0.465 |
| L PreCuneus | 7.5 (0.8) | 7.5 (0.8) | 7.6 (0.7) | 0.435 | 7.4 (1.0) | 7.5 (1.0) | 7.3 (1.0) | 0.318 | 0.424 | 0.833 | 0.138 |
| R PreCuneus | 7.7 (0.8) | 7.8 (0.8) | 7.6 (0.7) | 0.283 | 7.5 (0.9) | 7.6 (0.9) | 7.2 (1.0) | 0.097 | 0.242 | 0.497 | 0.142 |
| **Occipital** | | | | | | | | | | | |
| L Sup Occi Gy | 4.2 (0.5) | 4.3 (0.4) | 4.2 (0.5) | 0.517 | 4.2 (0.5) | 4.3 (0.5) | 4.1 (0.5) | 0.257 | 0.819 | 0.959 | 0.522 |
| R Sup Occi Gy | 4.7 (0.5) | 4.6 (0.5) | 4.7 (0.5) | 0.876 | 4.6 (0.6) | 4.7 (0.5) | 4.5 (0.7) | 0.323 | 0.595 | 0.889 | 0.313 |
| L Mid Occi Gy | 12.5 (1.1) | 12.5 (1.1) | 12.5 (1.2) | 0.915 | 12.5 (1.5) | 12.7 (1.5) | 12.2 (1.5) | 0.264 | 0.970 | 0.603 | 0.396 |
| R Mid Occi Gy | 12.3 (1.0) | 12.0 (1.0) | 12.6 (1.0) | 0.028 | 12.2 (1.4) | 12.2 (1.3) | 12.2 (1.6) | 0.825 | 0.582 | 0.458 | 0.265 |
| L Inf Occi Gy | 6.8 (0.7) | 6.9 (0.8) | 6.8 (0.6) | 0.707 | 6.8 (0.8) | 6.9 (0.8) | 6.7 (0.8) | 0.378 | 0.870 | 0.969 | 0.572 |
| R Inf Occi Gy | 6.8 (0.6) | 6.7 (0.6) | 6.8 (0.6) | 0.287 | 6.6 (0.8) | 6.6 (0.7) | 6.5 (0.8) | 0.748 | 0.129 | 0.631 | 0.151 |
| L Cuneus | 3.7 (0.4) | 3.7 (0.5) | 3.7 (0.4) | 0.711 | 3.7 (0.5) | 3.7 (0.5) | 3.7 (0.5) | 0.999 | 0.906 | 0.785 | 0.948 |
| R Cuneus | 4.1 (0.5) | 4.0 (0.5) | 4.1 (0.4) | 0.567 | 4.0 (0.5) | 4.0 (0.5) | 4.1 (0.6) | 0.728 | 0.689 | 0.879 | 0.844 |
| **Temporal** | | | | | | | | | | | |
| L Sup Temp Gy | 17.4 (1.4) | 17.4 (1.4) | 17.5 (1.3) | 0.782 | 17.4 (1.5) | 17.5 (1.5) | 17.1 (1.7) | 0.346 | 0.764 | 0.746 | 0.362 |
| R Sup Temp Gy | 16.7 (1.3) | 16.5 (1.3) | 16.8 (1.3) | 0.413 | 16.5 (1.4) | 16.6 (1.4) | 16.1 (1.5) | 0.205 | 0.317 | 0.822 | 0.085 |
| L Mid Temp Gy | 15.4 (1.3) | 15.4 (1.3) | 15.4 (1.4) | 0.899 | 15.4 (1.5) | 15.6 (1.4) | 14.9 (1.6) | 0.110 | 0.812 | 0.629 | 0.244 |
| R Mid Temp Gy | 15.6 (1.4) | 15.5 (1.3) | 15.7 (1.4) | 0.573 | 15.6 (1.6) | 15.6 (1.5) | 15.2 (1.8) | 0.415 | 0.588 | 0.797 | 0.301 |
| L Inf Temp Gy | 13.4 (1.1) | 13.4 (1.1) | 13.3 (1.1) | 0.731 | 13.2 (1.2) | 13.3 (1.1) | 13.0 (1.2) | 0.233 | 0.455 | 0.805 | 0.262 |
| R Inf Temp Gy | 14.0 (1.1) | 14.1 (1.2) | 14.0 (1.0) | 0.515 | 14.1 (1.3) | 14.1 (1.4) | 13.9 (1.3) | 0.592 | 0.854 | 0.974 | 0.985 |
| L ParaHipp Gy | 4.1 (0.3) | 4.1 (0.3) | 4.1 (0.3) | 0.988 | 4.1 (0.3) | 4.1 (0.3) | 4.1 (0.3) | 0.569 | 0.432 | 0.706 | 0.393 |
| R ParaHipp Gy | 4.4 (0.3) | 4.4 (0.3) | 4.3 (0.3) | 0.280 | 4.3 (0.4) | 4.4 (0.4) | 4.3 (0.4) | 0.511 | 0.736 | 0.603 | 0.782 |
| L Lingual Gy | 8.8 (0.8) | 8.9 (0.8) | 8.7 (0.8) | 0.383 | 8.6 (0.9) | 8.6 (0.9) | 8.6 (1.0) | 0.984 | 0.234 | 0.187 | 0.657 |
| R Lingual Gy | 8.6 (0.7) | 8.6 (0.6) | 8.6 (0.8) | 0.849 | 8.4 (0.9) | 8.4 (0.9) | 8.4 (1.0) | 0.989 | 0.250 | 0.305 | 0.550 |
| L Fusiform Gy | 8.1 (0.7) | 8.1 (0.7) | 8.1 (0.7) | 0.671 | 8.0 (0.7) | 8.0 (0.7) | 7.9 (0.6) | 0.359 | 0.308 | 0.534 | 0.262 |
| R Fusiform Gy | 7.8 (0.6) | 7.9 (0.7) | 7.7 (0.6) | 0.249 | 7.7 (0.8) | 7.8 (0.8) | 7.5 (0.6) | 0.218 | 0.252 | 0.376 | 0.204 |
| **Insula** | | | | | | | | | | | |
| L Insular Cortex | 5.9 (0.5) | 5.9 (0.5) | 5.9 (0.5) | 0.953 | 5.9 (0.5) | 5.9 (0.5) | 5.8 (0.5) | 0.798 | 0.430 | 0.593 | 0.533 |
| R Insular Cortex | 5.8 (0.5) | 5.8 (0.5) | 5.8 (0.5) | 0.940 | 5.8 (0.5) | 5.8 (0.5) | 5.8 (0.5) | 0.970 | 0.562 | 0.680 | 0.719 |
| **Limbic** | | | | | | | | | | | |
| L Cing Gy | 8.1 (0.8) | 8.1 (0.8) | 8.2 (0.9) | 0.879 | 8.0 (0.8) | 8.1 (0.7) | 7.9 (0.9) | 0.401 | 0.376 | 0.810 | 0.276 |
| R Cing Gy | 9.1 (0.8) | 9.1 (0.8) | 9.0 (0.8) | 0.466 | 9.1 (0.9) | 9.1 (0.9) | 8.9 (1.0) | 0.372 | 0.962 | 0.978 | 0.709 |
| L Hipp | 3.8 (0.3) | 3.8 (0.3) | 3.8 (0.3) | 0.410 | 3.8 (0.3) | 3.8 (0.3) | 3.8 (0.3) | 0.834 | 0.951 | 0.606 | 0.635 |
| R Hipp | 3.9 (0.3) | 3.9 (0.3) | 3.9 (0.3) | 0.598 | 3.9 (0.3) | 3.9 (0.3) | 3.9 (0.3) | 0.649 | 0.983 | 0.635 | 0.614 |
| **Caudate** | | | | | | | | | | | |
| L Caudate | 3.6 (0.4) | 3.7 (0.4) | 3.5 (0.4) | 0.049 | 3.6 (0.4) | 3.6 (0.4) | 3.5 (0.4) | 0.774 | 0.773 | 0.241 | 0.645 |
| R Caudate | 3.4 (0.4) | 3.4 (0.4) | 3.3 (0.4) | 0.046 | 3.3 (0.4) | 3.3 (0.4) | 3.3 (0.3) | 0.756 | 0.680 | 0.203 | 0.695 |
| **Putamen** | | | | | | | | | | | |
| L Putamen | 3.8 (0.6) | 3.7 (0.6) | 3.9 (0.6) | 0.217 | 3.6 (0.5) | 3.5 (0.5) | 3.7 (0.5) | 0.312 | 0.037 | 0.257 | 0.182 |
| R Putamen | 3.8 (0.5) | 3.7 (0.5) | 3.8 (0.5) | 0.269 | 3.6 (0.5) | 3.6 (0.5) | 3.7 (0.5) | 0.526 | 0.115 | 0.426 | 0.298 |
| **Cerebellum** | | | | | | | | | | | |
| BiLat Cerebellum | 86.2 (7.4) | 87.3 (7.2) | 85.2 (7.4) | 0.212 | 84.9 (8.2) | 84.7 (8.9) | 85.3 (6.7) | 0.736 | 0.328 | 0.147 | 0.944 |
| **Brainstem** | | | | | | | | | | | |
| BiLat Brainstem | 1.1 (0.2) | 1.2 (0.2) | 1.1 (0.2) | 0.024 | 1.2 (0.2) | 1.2 (0.2) | 1.1 (0.2) | **0.002*** | 0.571 | 0.724 | 0.436 |

Unit: cm^3^

mean (standard deviation)

Bonferroni methods were applied for multiple comparison correction; therefore, **P* value < 0.05/*k* in which *k* is the number of regions of interest within lobes/systems was considered statistically significant for regional brain volume between groups as indicated.

**Supplementary Table 4 Cortical thickness of different regions of brain**

|  | PCOS | | | | Control | | | | P value of PCOS v.s. Control | | |
| --- | --- | --- | --- | --- | --- | --- | --- | --- | --- | --- | --- |
|  | All  (n=76) | BMI < 25 Kg/m^2^  (n=35) | BMI≥25 Kg/m^2^  (n=41) | P value of BMI< and ≥25 Kg/m^2^ in PCOS | All  (n=68) | BMI < 25 Kg/m^2^  (n=45) | BMI≥25 Kg/m^2^  (n=23) | P value of BMI< and ≥25 Kg/m^2^ in Control | ALL | BMI<25 Kg/m^2^ | BMI≥25 Kg/m^2^ |
| **Frontal** | | | | | | | | | | | |
| L Cau Mid Fron | 2707.9 (111.1) | 2711.0 (117.5) | 2705.4 (106.8) | 0.831 | 2697.5 (113.8) | 2702.2 (108.9) | 2688.2 (124.7) | 0.649 | 0.579 | 0.735 | 0.581 |
| R Cau Mid Fron | 2718.3 (124.7) | 2734.6 (124.0) | 2704.3 (125.1) | 0.295 | 2691.1 (106.0) | 2694.7 (104.1) | 2684.0 (111.8) | 0.706 | 0.160 | 0.131 | 0.507 |
| L Lat OrbFron | 2718.8 (108.1) | 2721.9 (105.2) | 2716.2 (111.7) | 0.818 | 2701.4 (93.3) | 2705.8 (98.1) | 2692.9 (84.6) | 0.577 | 0.302 | 0.486 | 0.353 |
| R Lat OrbFron | 2682.3 (117.7) | 2688.3 (107.1) | 2677.2 (127.2) | 0.680 | 2653.2 (102.8) | 2664.6 (98.7) | 2630.9 (109.2) | 0.221 | 0.115 | 0.312 | 0.132 |
| L Med OrbFron | 2443.4 (107.5) | 2440.4 (104.4) | 2445.9 (111.4) | 0.826 | 2417.5 (121.6) | 2409.6 (125.0) | 2433.0 (115.8) | 0.447 | 0.181 | 0.234 | 0.667 |
| R Med OrbFron | 2490.0 (114.1) | 2494.6 (117.8) | 2486.0 (112.2) | 0.749 | 2478.1 (112.7) | 2477.4 (102.5) | 2479.6 (132.8) | 0.944 | 0.533 | 0.496 | 0.846 |
| L ParaCen | 2557.3 (100.8) | 2560.9 (108.1) | 2554.3 (95.4) | 0.780 | 2518.2 (99.9) | 2516.1 (94.3) | 2522.3 (112.4) | 0.823 | 0.021 | 0.056 | 0.256 |
| R ParaCen | 2510.1 (104.2) | 2511.7 (107.0) | 2508.7 (103.0) | 0.902 | 2491.8 (103.2) | 2492.8 (94.9) | 2489.8 (120.0) | 0.916 | 0.292 | 0.414 | 0.528 |
| L Pars Oper | 2766.0 (97.9) | 2767.5 (103.3) | 2764.8 (94.4) | 0.906 | 2760.0 (100.9) | 2770.1 (97.7) | 2740.4 (106.5) | 0.271 | 0.718 | 0.911 | 0.366 |
| R Pars Oper | 2776.6 (112.7) | 2778.3 (113.7) | 2775.1 (113.3) | 0.902 | 2736.5 (92.3) | 2738.6 (84.6) | 2732.5 (107.5) | 0.815 | 0.021 | 0.089 | 0.143 |
| L Pars Orb | 2786.5 (127.1) | 2780.8 (141.0) | 2791.4 (115.5) | 0.725 | 2746.4 (134.4) | 2755.7 (124.4) | 2728.0 (153.4) | 0.459 | 0.068 | 0.409 | 0.093 |
| R Pars Orb | 2709.2 (130.3) | 2720.8 (138.2) | 2699.4 (124.0) | 0.483 | 2681.8 (137.0) | 2700.2 (133.4) | 2645.9 (139.8) | 0.131 | 0.222 | 0.504 | 0.134 |
| L Pars Tri | 2689.8 (102.1) | 2682.9 (95.8) | 2695.7 (108.0) | 0.587 | 2673.8 (124.4) | 2676.1 (139.1) | 2669.4 (91.8) | 0.815 | 0.404 | 0.795 | 0.308 |
| R Pars Tri | 2676.7 (104.8) | 2665.8 (111.4) | 2686.0 (99.2) | 0.410 | 2639.7 (103.8) | 2638.8 (108.9) | 2641.3 (95.1) | 0.923 | 0.035 | 0.281 | 0.082 |
| L PreCen | 2565.6 (111.2) | 2566.1 (123.3) | 2565.2 (101.3) | 0.974 | 2554.9 (98.1) | 2554.6 (91.7) | 2555.7 (111.8) | 0.967 | 0.542 | 0.646 | 0.737 |
| R PreCen | 2533.7 (112.9) | 2547.6 (119.3) | 2521.9 (107.2) | 0.329 | 2510.4 (102.7) | 2507.1 (86.1) | 2516.7 (131.4) | 0.754 | 0.196 | 0.096 | 0.873 |
| L Ros Mid Fron | 2528.3 (85.7) | 2519.1 (93.0) | 2536.2 (79.2) | 0.394 | 2512.9 (93.1) | 2520.4 (96.3) | 2498.4 (86.7) | 0.347 | 0.306 | 0.952 | 0.092 |
| R Ros Mid Fron | 2511.2 (94.1) | 2501.1 (92.0) | 2519.8 (96.1) | 0.390 | 2488.2 (79.7) | 2489.6 (84.6) | 2485.6 (70.7) | 0.838 | 0.114 | 0.565 | 0.109 |
| L Sup Fron | 2898.1 (105.7) | 2903.2 (119.3) | 2893.7 (93.8) | 0.707 | 2887.1 (102.6) | 2892.2 (105.2) | 2876.9 (99.0) | 0.557 | 0.527 | 0.671 | 0.510 |
| R Sup Fron | 2905.9 (101.1) | 2913.0 (120.9) | 2899.9 (81.7) | 0.589 | 2895.3 (98.2) | 2900.2 (105.8) | 2885.7 (82.6) | 0.535 | 0.525 | 0.623 | 0.510 |
| L Fron Pole | 2711.3 (232.7) | 2701.5 (227.2) | 2719.7 (239.7) | 0.735 | 2648.5 (171.4) | 2649.4 (187.9) | 2646.7 (137.5) | 0.946 | 0.065 | 0.277 | 0.127 |
| R Fron Pole | 2637.8 (198.0) | 2619.2 (177.6) | 2653.7 (215.2) | 0.447 | 2600.7 (189.8) | 2563.8 (193.0) | 2673.0 (164.0) | 0.018 | 0.253 | 0.186 | 0.688 |
| **Parietal** | | | | | | | | | | | |
| L Inf Parie | 2497.5 (88.1) | 2488.1 (78.4) | 2505.6 (95.9) | 0.382 | 2487.7 (82.6) | 2488.8 (83.9) | 2485.5 (82.0) | 0.877 | 0.489 | 0.968 | 0.379 |
| R Inf Parie | 2477.6 (93.5) | 2466.4 (81.8) | 2487.1 (102.4) | 0.333 | 2454.4 (79.7) | 2455.7 (82.8) | 2452.0 (74.9) | 0.854 | 0.111 | 0.564 | 0.122 |
| L PostCen | 2260.2 (90.9) | 2232.6 (88.7) | 2283.6 (87.1) | 0.014 | 2226.7 (87.6) | 2220.5 (84.6) | 2238.9 (93.8) | 0.433 | 0.026 | 0.536 | 0.067 |
| R PostCen | 2266.9 (89.0) | 2252.5 (86.8) | 2279.2 (90.0) | 0.192 | 2239.6 (86.8) | 2246.7 (79.2) | 2225.6 (100.4) | 0.387 | 0.064 | 0.759 | 0.039 |
| L PreCuneus | 2449.9 (86.0) | 2449.7 (84.4) | 2450.1 (88.4) | 0.986 | 2425.2 (87.6) | 2420.2 (91.0) | 2435.0 (81.6) | 0.500 | 0.091 | 0.138 | 0.495 |
| R PreCuneus | 2465.2 (86.1) | 2471.4 (78.8) | 2460.0 (92.6) | 0.563 | 2442.7 (90.1) | 2438.0 (89.2) | 2452.1 (93.2) | 0.553 | 0.129 | 0.080 | 0.746 |
| L Sup Parie | 2310.9 (72.8) | 2296.8 (65.1) | 2323.0 (77.5) | 0.114 | 2281.2 (78.1) | 2278.9 (82.2) | 2285.8 (71.0) | 0.721 | 0.020 | 0.280 | 0.058 |
| R Sup Parie | 2321.7 (79.9) | 2315.5 (73.2) | 2327.0 (85.7) | 0.530 | 2278.1 (76.6) | 2280.0 (82.6) | 2274.5 (64.9) | 0.766 | **0.001*** | 0.045 | **0.008*** |
| L SupraMar | 2590.3 (93.8) | 2586.4 (93.3) | 2593.6 (95.3) | 0.741 | 2560.5 (83.0) | 2560.0 (85.5) | 2561.4 (79.7) | 0.947 | 0.045 | 0.197 | 0.155 |
| R SupraMar | 2581.9 (94.9) | 2568.6 (100.3) | 2591.7 (89.9) | 0.297 | 2540.7 (86.8) | 2547.2 (85.6) | 2527.9 (89.7) | 0.398 | **0.009*** | 0.317 | **0.009*** |
| **Occipital** | | | | | | | | | | | |
| L Cuneus | 2085.0 (95.2) | 2071.8 (94.3) | 2096.2 (95.7) | 0.268 | 2059.9 (98.3) | 2058.6 (101.2) | 2062.4 (94.7) | 0.878 | 0.123 | 0.549 | 0.179 |
| R Cuneus | 2065.4 (78.0) | 2057.9 (78.9) | 2071.8 (77.6) | 0.441 | 2050.9 (98.7) | 2045.2 (106.7) | 2062.1 (81.7) | 0.473 | 0.333 | 0.543 | 0.642 |
| L Lat Occi | 2127.3 (77.8) | 2117.6 (67.6) | 2135.6 (85.6) | 0.310 | 2120.5 (79.3) | 2114.1 (83.1) | 2132.9 (71.2) | 0.334 | 0.604 | 0.835 | 0.896 |
| R Lat Occi | 2119.9 (82.4) | 2107.8 (90.4) | 2130.2 (74.5) | 0.248 | 2115.9 (78.2) | 2125.016 (81.914) | 2098.2 (68.5) | 0.160 | 0.771 | 0.382 | 0.089 |
| L Lingual | 2077.5 (86.5) | 2087.8 (97.5) | 2068.8 (76.0) | 0.352 | 2068.6 (90.6) | 2074.9 (95.6) | 2056.5 (80.7) | 0.408 | 0.548 | 0.554 | 0.553 |
| R Lingual | 2086.7 (82.7) | 2089.5 (84.2) | 2084.3 (82.3) | 0.789 | 2074.4 (84.5) | 2074.0 (80.9) | 2075.0 (93.0) | 0.965 | 0.378 | 0.410 | 0.691 |
| L PeriCal | 2019.5 (111.6) | 2026.8 (127.8) | 2013.3 (96.8) | 0.611 | 2009.7 (107.9) | 2003.5 (107.5) | 2021.9 (110.0) | 0.514 | 0.594 | 0.390 | 0.756 |
| R PeriCal | 2033.3 (111.2) | 2026.3 (121.7) | 2039.3 (102.5) | 0.621 | 2021.0 (112.1) | 2020.3 (113.5) | 2022.5 (111.7) | 0.938 | 0.511 | 0.821 | 0.556 |
| **Temporal** | | | | | | | | | | | |
| L Banks STS | 2530.5 (139.8) | 2531.6 (132.4) | 2529.5 (147.5) | 0.950 | 2539.7 (142.3) | 2535.8 (127.6) | 2547.5 (170.3) | 0.771 | 0.694 | 0.887 | 0.672 |
| R Banks STS | 2656.5 (155.3) | 2639.1 (130.2) | 2671.4 (174.2) | 0.358 | 2637.8 (125.7) | 2620.9 (135.8) | 2670.8 (97.6) | 0.087 | 0.425 | 0.545 | 0.985 |
| L Entorhinal | 3147.8 (390.7) | 3141.5 (402.0) | 3153.1 (385.6) | 0.898 | 3087.7 (363.0) | 3042.9 (397.1) | 3175.5 (271.5) | 0.111 | 0.341 | 0.277 | 0.787 |
| R Entorhinal | 3278.2 (366.7) | 3255.2 (407.7) | 3297.8 (331.6) | 0.623 | 3206.7 (348.8) | 3180.5 (359.5) | 3258.0 (328.6) | 0.377 | 0.233 | 0.395 | 0.645 |
| L Fusiform | 2489.3 (98.0) | 2478.9 (96.6) | 2498.1 (99.6) | 0.398 | 2463.5 (95.5) | 2467.0 (90.0) | 2456.7 (107.2) | 0.695 | 0.112 | 0.573 | 0.135 |
| R Fusiform | 2490.0 (107.7) | 2455.0 (97.9) | 2519.8 (107.8) | **0.008*** | 2474.9 (97.3) | 2466.0 (94.7) | 2492.3 (102.0) | 0.309 | 0.378 | 0.615 | 0.315 |
| L Inf Temp Gy | 2607.9 (121.9) | 2591.2 (132.1) | 2622.1 (112.1) | 0.280 | 2586.7 (104.5) | 2585.2 (106.0) | 2589.6 (103.7) | 0.869 | 0.263 | 0.826 | 0.248 |
| R Inf Temp Gy | 2614.9 (126.9) | 2611.8 (133.7) | 2617.6 (122.5) | 0.846 | 2584.7 (96.6) | 2590.5 (102.2) | 2573.2 (85.5) | 0.464 | 0.108 | 0.439 | 0.095 |
| L Mid Temp Gy | 2816.8 (124.7) | 2795.0 (124.5) | 2835.5 (123.3) | 0.160 | 2790.3 (100.0) | 2789.1 (92.0) | 2792.6 (116.1) | 0.898 | 0.159 | 0.816 | 0.173 |
| R Mid Temp Gy | 2869.3 (134.4) | 2849.6 (134.2) | 2886.2 (133.8) | 0.239 | 2815.4 (95.0) | 2811.1 (95.7) | 2823.7 (95.2) | 0.610 | **0.006*** | 0.156 | 0.034 |
| L ParaHipp | 2423.6 (210.9) | 2409.3 (239.4) | 2435.8 (185.2) | 0.597 | 2405.6 (175.9) | 2404.3 (158.7) | 2408.1 (209.4) | 0.940 | 0.576 | 0.915 | 0.599 |
| R ParaHipp | 2457.8 (197.5) | 2441.8 (226.5) | 2471.5 (170.6) | 0.526 | 2451.2 (195.7) | 2462.4 (203.8) | 2429.1 (181.1) | 0.496 | 0.839 | 0.674 | 0.364 |
| L Sup Temp Gy | 2725.6 (133.0) | 2719.9 (143.5) | 2730.5 (124.9) | 0.734 | 2703.2 (120.8) | 2701.2 (127.6) | 2707.2 (109.0) | 0.840 | 0.291 | 0.546 | 0.440 |
| R Sup Temp Gy | 2798.0 (144.3) | 2786.2 (149.2) | 2808.0 (141.1) | 0.516 | 2769.2 (97.2) | 2760.5 (98.6) | 2786.4 (94.3) | 0.297 | 0.160 | 0.383 | 0.466 |
| L Temp Pole | 3350.8 (299.2) | 3331.5 (337.0) | 3367.2 (266.0) | 0.615 | 3344.8 (229.4) | 3319.5 (234.8) | 3394.3 (214.9) | 0.195 | 0.893 | 0.858 | 0.660 |
| R Temp Pole | 3440.1 (262.5) | 3441.3 (264.4) | 3439.0 (264.2) | 0.970 | 3415.8 (202.6) | 3396.3 (207.5) | 3453.8 (191.4) | 0.261 | 0.533 | 0.411 | 0.797 |
| L Trans Temp | 2577.0 (111.1) | 2574.3 (126.6) | 2579.2 (97.5) | 0.853 | 2576.1 (132.1) | 2577.3 (130.4) | 2573.5 (138.4) | 0.913 | 0.964 | 0.918 | 0.863 |
| R Trans Temp | 2590.1 (153.7) | 2576.2 (182.3) | 2602.0 (125.4) | 0.482 | 2576.4 (150.5) | 2573.8 (149.1) | 2581.5 (156.5) | 0.847 | 0.590 | 0.950 | 0.593 |
| **Cingulate** | | | | | | | | | | | |
| L Cau Ant Cing | 2598.3 (200.0) | 2568.6 (210.2) | 2623.7 (189.7) | 0.238 | 2527.9 (220.3) | 2546.7 (226.4) | 2491.0 (207.8) | 0.316 | 0.047 | 0.656 | 0.015 |
| R Cau Ant Cing | 2526.9 (174.7) | 2524.0 (137.8) | 2529.4 (202.7) | 0.890 | 2561.9 (188.1) | 2541.1 (211.5) | 2602.5 (125.3) | 0.139 | 0.252 | 0.663 | 0.080 |
| L Isthmus Cing | 2071.2 (176.4) | 2048.4 (178.3) | 2090.7 (174.6) | 0.302 | 2080.1 (178.7) | 2053.2 (196.1) | 2132.6 (126.3) | 0.048 | 0.767 | 0.910 | 0.273 |
| R Isthmus Cing | 2134.2 (123.9) | 2107.4 (133.3) | 2156.9 (111.8) | 0.087 | 2130.4 (134.1) | 2125.8 (131.4) | 2139.5 (141.9) | 0.701 | 0.862 | 0.542 | 0.614 |
| L Post Cing | 2334.5 (121.1) | 2358.6 (131.9) | 2313.9 (108.4) | 0.115 | 2297.7 (126.7) | 2273.5 (110.9) | 2345.1 (144.2) | 0.044 | 0.078 | **0.003*** | 0.372 |
| R Post Cing | 2333.5 (96.3) | 2352.1 (96.9) | 2317.6 (94.1) | 0.122 | 2331.0 (123.1) | 2314.0 (132.1) | 2364.3 (97.3) | 0.081 | 0.893 | 0.141 | 0.069 |
| L Ros Ant Cing | 2549.9 (143.6) | 2556.4 (147.8) | 2544.3 (141.5) | 0.717 | 2519.3 (180.5) | 2530.4 (185.5) | 2497.5 (172.1) | 0.471 | 0.266 | 0.487 | 0.274 |
| R Ros Ant Cing | 2650.1 (175.5) | 2685.5 (184.8) | 2619.9 (163.3) | 0.108 | 2620.7 (166.6) | 2646.5 (166.0) | 2570.4 (159.2) | 0.073 | 0.305 | 0.331 | 0.243 |
| **Insula** | | | | | | | | | | | |
| L Insula | 2989.5 (136.2) | 3003.0 (120.5) | 2978.0 (148.9) | 0.422 | 2993.6 (148.4) | 2977.0 (145.4) | 3025.9 (152.0) | 0.210 | 0.866 | 0.386 | 0.230 |
| R Insula | 2945.0 (153.9) | 2961.1 (160.8) | 2931.3 (148.3) | 0.407 | 2939.4 (179.6) | 2909.3 (169.0) | 2998.2 (188.8) | 0.064 | 0.841 | 0.167 | 0.151 |

Unit: 10^-3^ mm

mean (standard deviation)

Bonferroni methods were applied for multiple comparison correction; therefore, **P* value < 0.05/*k* in which *k* is the number of regions of interest within lobes/systems was considered statistically significant for regional brain volume between groups as indicated.
